# Synergistic dual chemophysical FeCu-MOF scaffold with PEMF stimulation drives angiogenic-osteogenic coupling for bone regeneration

**DOI:** 10.1016/j.mtbio.2025.102324

**Published:** 2025-09-18

**Authors:** Dongdong Guo, Wenjie Wang, Dongyang Zhao, Tianyu Chen, Xingyu Ma, Yixiao Li, Xiaojun Zhang

**Affiliations:** a Department of Orthopedics, Northwest University First Hospital, Xi'an, Shaanxi Province, 710043, China; b Key Laboratory of Resource Biology and Biotechnology in Western China, Ministry of Education, Provincial Key Laboratory of Biotechnology, College of Life Sciences, Northwest University, Xi'an, Shaanxi Province, 710069, China; c School of Medicine, Northwest University, Xi'an, Shaanxi Province, 710069, China

**Keywords:** FeCu-MOF, Pulsed electromagnetic fields (PEMF), Chemophysical synergy, Angiogenesis, Osteogenesis, Bone regeneration

## Abstract

Repairing large bone defects effectively requires concurrent osteogenesis and angiogenesis, a significant challenge for conventional biomaterials often limited by suboptimal structural design and an inability to provide spatiotemporally controlled bioactive cues. Here, we report a novel chemophysical dual-responsive system rationally designed to address this osteogenic-angiogenic coupling challenge. This system integrates a structurally engineered bimetallic FeCu-metal-organic framework (FeCu-MOF) within a poly(lactic acid)/hydroxyapatite (PLA/HA) scaffold. The engineered FeCu-MOF architecture enables the programmed and sustained co-release of Fe^3+^ and Cu^2+^ ions, providing tailored chemical signals. Synergistic pulsed electromagnetic field (PEMF) stimulation was introduced as a physical cue to further enhance the scaffold's bioactivity. The composite scaffolds, featuring interconnected hierarchical porosity and enhanced hydrophilicity due to FeCu-MOF incorporation, demonstrated distinct Fe^3+^/Cu^2+^ release profiles. *In vitro*, these scaffolds exhibited excellent biocompatibility and significantly promoted bone marrow mesenchymal stem cells (BMSCs) proliferation and osteogenic differentiation. Notably, this structure-derived dual-ion release also indicated pro-angiogenic potential. Crucially, daily PEMF treatment synergistically amplified these cellular responses. *In vivo* evaluation in a rat cranial defect model confirmed the system's efficacy. While FeCu-MOF/PLA/HA scaffolds alone enhanced bone regeneration, their combination with PEMF yielded the most robust outcomes, characterized by markedly superior vascularized bone formation. Comprehensive analysis, including micro-CT, histology, and immunohistochemistry, confirmed these findings by demonstrating improved bone volume, density, and architecture, mature integrated tissue, and enhanced coupled expression of CD31 and osteogenic markers. In summary, the study validates a powerful synergistic strategy for enhanced bone regeneration. This strategy, integrating programmable, structure-derived bimetallic ion release with PEMF stimulation, successfully achieved synergistic angiogenic-osteogenic coupling, offering a promising approach for complex defect scenarios.

## Highlights

• Novel FeCu-MOF integrates chemical cues with PEMF physical stimuli.
• Engineered bimetallic MOF enables programmed Fe^3+^/Cu^2+^ co-release.
• Dual chemophysical system synergistically promotes angiogenic-osteogenic coupling.
• PEMF amplifies cellular responses to MOF-derived ionic signals.
• Marked vascularized bone regeneration in a cranial defect model.

## Graphical abstract

**Figure:**
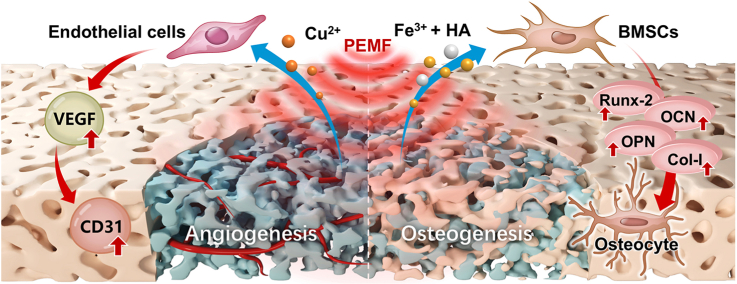

## 1 Introduction

Large bone defects, resulting from trauma, tumor resection, or congenital abnormalities, pose a significant clinical challenge due to the limited self-healing capacity of bone tissue [1,2]. While autografts remain the clinical gold standard, their application is hampered by donor site morbidity, limited availability, and potential complications [3,4]. Allografts and conventional synthetic implants often suffer from risks of immune rejection, infection, and inadequate integration with host tissue [5,6]. These limitations are partly attributed to their suboptimal structural biomimicry and limited control over the local biological microenvironment. To overcome these limitations, tissue engineering strategies focusing on the rational design of biomaterials have emerged as a promising alternative [7,8]. These strategies typically employ biodegradable scaffolds engineered with specific architectures and compositions to mimic the natural bone extracellular matrix and actively promote regeneration. However, achieving efficient and functional regeneration remains a major hurdle. This is particularly true for complex defects within compromised microenvironments that demand both rapid vascularization and robust osteogenesis [[9], [10], [11]]. A key challenge lies in designing scaffold structures that can effectively orchestrate these coupled biological processes.

To address these limitations, significant efforts focus on developing bioactive scaffolds with tailored structural and chemical properties designed to orchestrate the cellular and molecular events of bone repair [10,12,13]. Incorporating osteogenic growth factors or therapeutic ions (e.g., Fe^3+^, Sr^2+^, Mg^2+^, Cu^2+^) into scaffold matrices can enhance osteogenesis [14,15]. The rational design of systems for the programmed co-release of multiple ions has emerged as a particularly powerful strategy to orchestrate the complex interplay between different cellular processes. For example, the co-delivery of magnesium and silicon ions from engineered microcryogels was recently shown to effectively drive angiogenic-osteogenic coupling while concurrently creating a pro-regenerative immune microenvironment [16]. While such advanced systems demonstrate the immense potential of ion-based therapies, achieving precise spatiotemporal control over bioactive cues remains a central challenge in the field. Nevertheless, challenges such as burst release, short half-lives of growth factors, and difficulties in achieving sustained, localized delivery persist [17,18]. These issues often stem from limitations in current scaffold designs and drug delivery mechanisms. More critically, successful regeneration hinges on the concurrent promotion of angiogenesis and osteogenesis [19]. However, designing a single scaffold system capable of such coordinated delivery remains a significant challenge. Such a system must possess the structural attributes for effective and controllable co-delivery of both pro-osteogenic and pro-angiogenic cues in a temporal manner. This challenge is particularly acute in scenarios demanding rapid blood supply for large-volume bone formation. In such cases, the scaffold's architecture and material composition must synergistically support both vascular network formation and bone matrix deposition.

Iron (Fe^3+^) and copper (Cu^2+^) are bioactive ions attracting significant attention for their unique roles in bone metabolism and angiogenesis. Fe^3+^ is essential for cellular metabolism and also participates in regulating inflammatory responses and osteoblast function [20,21]. Conversely, Cu^2+^ is widely recognized as a potent pro-angiogenic factor, for instance, by stabilizing HIF-1α, and it can also stimulate collagen synthesis [14].We hypothesized that a biomaterial system, specifically engineered for the programmed and sustained co-release of Fe^3+^ and Cu^2+^, could effectively promote concurrent osteogenesis and angiogenesis. This approach aims to address the critical coupling challenge in bone regeneration. Metal-organic frameworks (MOFs) offer an ideal platform for such controlled co-release, primarily because their highly tunable porosity, exceptionally high surface area, and versatile coordination chemistry serve as key structural attributes for programmed ion delivery [22,23].

Building on this hypothesis, we synthesized and characterized a novel bimetallic FeCu-MOF. This MOF was specifically engineered with a tailored framework structure, leveraging its intrinsic porosity and metal-ligand coordination for the controlled co-release of Fe^3+^ and Cu^2+^ ions. This FeCu-MOF was subsequently integrated into a biocompatible poly(lactic acid)/hydroxyapatite (PLA/HA) composite matrix, forming a FeCu-MOF/PLA/HA scaffold. The design of this composite scaffold aimed to combine the FeCu-MOF's ion-releasing functionality with the PLA/HA matrix's favorable biocompatibility and osteoconductivity. The goal was to develop a system with intrinsic, chemically-induced regenerative capacity, marking its novel application in bone regeneration through combined chemophysical stimulation within a PLA/HA scaffold. Building on this, our study focused on elucidating how the system's structure-derived ion release profile synergistically modulates the local microenvironment.

Beyond chemical/biological signals, physical stimuli, such as pulsed electromagnetic fields (PEMF), are increasingly recognized as non-invasive modalities to modulate cellular behavior and promote bone healing [24,25]. PEMF, in particular, has demonstrated clinical benefits in treating non-unions and accelerating fracture repair, potentially by influencing ion transport, cell signaling pathways, and gene expression [24,26,27]. This led us to further hypothesize that integrating PEMF stimulation with our ion-releasing FeCu-MOF/PLA/HA scaffold could significantly amplify its therapeutic efficacy, thereby creating a chemophysical dual-responsive system. Despite the individual promise of bioactive scaffolds and physical therapies, a critical gap exists in designing integrated systems. Specifically, systems where the material's structural and chemical properties are rationally designed to synergize with external physical fields remain underdeveloped [28]. Current approaches often focus on either material-based bioactivity or responsiveness to physical stimuli, but typically lack the sophisticated structural and functional integration required for optimal synergistic effects.

**Scheme 1:**
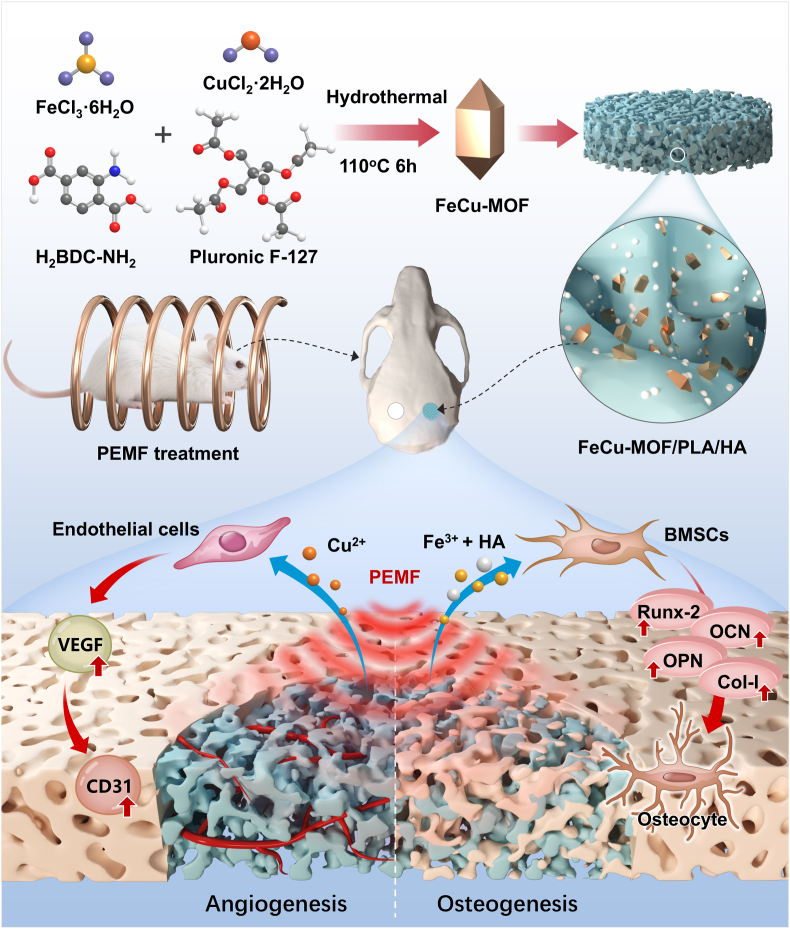
Schematic illustration of the synergistic dual chemophysical strategy for vascularized bone regeneration. (A) The process begins with the hydrothermal synthesis of bimetallic FeCu-MOF nanoparticles and their incorporation into a porous PLA/HA scaffold. (B) The resulting composite scaffold is implanted into a rat cranial defect and subjected to PEMF treatment. (C) This activates a dual-responsive mechanism, where the scaffold provides a programmed co-release of chemical cues: pro-angiogenic Cu2+ and pro-osteogenic Fe3+ ions. Working in concert with the physical stimulus (PEMF), the released Cu2+ ions stimulate endothelial cells to promote angiogenesis, as evidenced by VEGF and CD31 upregulation. Concurrently, Fe3+ ions and HA, also potentiated by PEMF, stimulate BMSCs to drive robust osteogenesis, as evidenced by the upregulation of Runx-2, OCN, OPN, and Col-I. This tightly coupled angiogenic-osteogenic response leads to effective and accelerated regeneration of vascularized bone tissue.

A next-generation bone graft substitute should ideally provide controlled release of multiple therapeutic agents from an engineered structural matrix and exhibit enhanced responsiveness to non-invasive physical interventions like PEMF to orchestrate complex biological processes [28,29]. However, limited research has addressed the rational design and evaluation of biomaterial systems that effectively integrate these chemophysical multi-modal synergistic approaches. Therefore, we were guided by a central hypothesis: a bimetallic FeCu-MOF, engineered for programmed Fe^3+^/Cu^2+^ co-release and integrated within a PLA/HA scaffold, would synergistically interact with PEMF stimulation to drive angiogenic-osteogenic coupling and enhance bone regeneration. Based on this hypothesis, this study aimed to construct and evaluate such a novel chemophysical dual-responsive system (Scheme 1). To test this hypothesis, our systematic design and characterization of the FeCu-MOF and the composite FeCu-MOF/PLA/HA scaffold focused on its structure-derived ion release kinetics and physical properties. We then evaluated *in vitro* cellular responses to the scaffold's chemical cues and the chemophysical synergy with PEMF. Ultimately, its *in vivo* regenerative efficacy, particularly the structure-function relationship in promoting vascularized bone formation, was validated in a cranial defect model. By integrating rationally designed material-based chemical signals with physical stimuli, this work aims to provide a new paradigm for developing highly effective bone repair strategies. If proven effective, this structurally engineered FeCu-MOF-based dual-responsive system offers a promising candidate for treating complex bone defects. Moreover, this study deepens our understanding of the intricate interplay between material structure, cellular responses, and physical field modulation in regenerative medicine.

## 2 Materials and methods

### 2.1 Materials

Iron(III) chloride hexahydrate (FeCl_3_·6H_2_O, ≥99 %), cupric(II) chloride dihydrate (CuCl_2_·2H_2_O, ≥99 %), 2-Aminoterephthalic acid (NH_2_-BDC, ≥98 %), nano-hydroxyapatite (nHA, particle size <60 nm), acetic acid (CH_3_COOH, glacial, ≥99.5 %), ethanol (reagent grade, ≥99.7 %), and dichloromethane (CH_2_Cl_2_, anhydrous, ≥99.8 %) were purchased from Macklin Biochemical Co., Ltd. (Shanghai, China). Pluronic® F-127 was obtained from the Beyotime Institute of Biotechnology (Shanghai, China). Sodium chloride (NaCl, ≥99.5 %) was received from Aladdin Biochemical Technology Co., Ltd. (Shanghai, China). Poly(lactic acid) (PLA, Evonik, Essen, Germany) was used as the base polymer. Fetal bovine serum (FBS) was purchased from cell box (Hong Kong, China). Minimum Essential Medium Alpha Modification (α-MEM), and Penicillin-Streptomycin solution (100X) were purchased from Gibco (Waltham, MA, USA). All cell culture reagents were of cell culture grade, and other chemicals were of analytical grade unless otherwise specified.

### 2.2 Preparation of the FeCu-MOF nanoparticles

FeCu-MOF, designed for the co-delivery of Fe^3+^ and Cu^2+^ ions, was synthesized via solvothermal method according to previous report [30,31]. Briefly, 0.16 g of Pluronic® F-127 was dissolved in 13.34 mL of deionized water (Solution A). Separately, 0.18 g of FeCl_3_·6H_2_O and 0.08 g of CuCl_2_·2H_2_O were dissolved in 1.66 mL of deionized water (Solution B). Under magnetic stirring, Solution B was added dropwise to Solution A and stirred for 1 h. Subsequently, 0.3 mL of glacial acetic acid was added, and stirring continued for another 1 h. Finally, 60 mg of NH_2_-BDC ligand was added, and the mixture was stirred for an additional 2 h. The resulting solution was transferred to a Teflon-lined stainless-steel autoclave and heated at 110 °C for 16 h. After cooling to room temperature, the brownish-yellow solid product was collected by centrifugation (11000 rpm, 15 min). The product was then washed thoroughly three times each with deionized water and absolute ethanol to remove unreacted precursors and surfactant. The final FeCu-MOF powder was dried under vacuum at 60 °C for 12 h.

### 2.3 Preparation of FeCu-MOF/PLA/HA composite porous scaffolds

The FeCu-MOF/PLA/HA composite porous scaffolds, designed to provide a three-dimensional matrix for cell growth and controlled ion delivery, were fabricated through a modified solvent casting-particulate leaching technique [32]. Sodium chloride (NaCl) particles as the porogenic agent to create an interconnected porous network. The compositions of different FeCu-MOF/PLA/HA scaffolds, varied to investigate the dose-dependent effects of FeCu-MOF, are listed in Table 1.

**Table 1:** Ingredients of different FeCu-MOF/PLA/HA scaffolds.

Group	PLA Concentration (g/ml)	FeCu-MOF/PLA (wt/wt)	HA/PLA (wt/wt)	NaCl/PLA (wt/wt)
PLA/HA	0.1	0 %	20 %	9
2 FeCu-MOF/PLA/HA	0.1	2 %	20 %	9
4 FeCu-MOF/PLA/HA	0.1	4 %	20 %	9
8 FeCu-MOF/PLA/HA	0.1	8 %	20 %	9

Based on established literature on the bioactive concentrations of copper and iron ions and on our preliminary screening, a range of 2 %, 4 %, and 8 % (wt/wt) FeCu-MOF loading was selected to systematically investigate the dose-dependent effects and identify an optimal therapeutic window. PLA was dissolved in dichloromethane (CH_2_Cl_2_) to prepare a 10 % (w/v) solution. For the PLA/HA group, 0.14 g of nHA was dispersed in 7 mL of the 10 % PLA solution using magnetic stirring until visually homogeneous. For the composite groups, designated amounts of FeCu-MOF (corresponding to 2 %, 4 %, or 8 % wt/wt relative to PLA, as detailed in Table 1) were added to the PLA/nHA suspension. The mixture was then continuously stirred for 12 h and subsequently ultrasonicated for 30 min to ensure uniform dispersion. NaCl particles (sieved to 200–400 μm) were added to the polymer/MOF suspension at a NaCl:PLA weight ratio of 9:1. The mixture was subsequently stirred for 30 min to achieve a uniform slurry. The resulting slurry was cast into a 75 mm diameter polystyrene Petri dish and air-dried in a fume hood for 12 h. After solidification, the composite was demolded. It was then immersed in deionized water for 72 h under gentle stirring to leach out the NaCl porogen, with the water being replaced approximately every 6 h. Finally, the resulting porous scaffolds were air-dried at room temperature and cut into discs (Ø 10 mm, ∼1 mm thickness) for subsequent experiments. The mild conditions of this leaching step ensure the structural stability of the embedded FeCu-MOF. The PLA matrix acts as a diffusion barrier, preventing any significant premature loss of ions during fabrication.

### 2.4 Characterization of FeCu-MOF nanoparticles

The morphology and structure of the synthesized FeCu-MOF were characterized by scanning electron microscopy (SEM; JSM-7610FPlus, JEOL, Tokyo, Japan), operating at 15 kV. Transmission electron microscopy (TEM; JEM-F200, JEOL, Tokyo, Japan), operating at 200 kV, was used to further observe nanoparticle morphology and internal structure. Energy-dispersive X-ray spectroscopy (EDS) coupled with TEM was used to determine the elemental composition and distribution within the nanoparticles. The hydrodynamic size distribution in aqueous dispersion was measured by dynamic light scattering (DLS; Zetasizer Nano ZS90, Malvern Panalytical, Malvern, UK) at 25 °C. The crystalline structure was analyzed by X-ray powder diffraction (XRD; Smart Lab SE diffractometer, Rigaku, Kyoto, Japan) using Cu Kα radiation (λ = 1.54 Å) over a 2θ range of 5–25° at a scan rate of 2°/min. Fourier Transform Infrared Spectroscopy (FTIR) was performed using a spectrometer (Thermo Fisher Scientific, Waltham, MA, USA) in the range of 4000–400 cm^−1^ with KBr pellets.

### 2.5 Characterization of FeCu-MOF/PLA/HA composite porous scaffolds

The surface morphology, porous architecture, and elemental distribution of the scaffolds were observed by SEM (JSM-7610FPlus; JEOL, Tokyo, Japan) after sputter-coating with gold. Surface hydrophilicity was assessed by measuring the static water contact angle using an OCA25 contact angle goniometer (DataPhysics Instruments, Filderstadt, Germany). A 20 μL droplet of deionized water was placed onto the scaffold surface, and contact angles were recorded 10 s after deposition. The release profiles of Fe^3+^ and Cu^2+^ ions from the composite scaffolds were determined by inductively coupled plasma mass spectrometry (ICP-MS; ICPMS-2030, Shimadzu, Kyoto, Japan). For ion release studies, scaffold discs (n = 3 per group) were immersed in 5 mL of phosphate-buffered saline (PBS, pH 7.4) at 37 °C under gentle shaking. At predetermined time points (0, 1, 4, 7, 14, 21, and 28 days), the supernatant was collected. An equal volume of fresh PBS was added to the remaining solution, and the collected samples were diluted appropriately. Fe and Cu concentrations in the diluted samples were then analyzed by ICP-MS against standard calibration curves.

### 2.6 *In vitro* cell experiments

#### 2.6.1 Cell culture

Primary rat bone marrow-derived mesenchymal stem cells (BMSCs) were isolated from the femurs and tibias of 1-week-old male Sprague-Dawley (SD) rats (Experimental Animal Centre, Xi'an Jiaotong University, Shanxi, China) according to established protocols [33]. Briefly, bone marrow was flushed from the femurs and tibias using α-MEM (Gibco), then collected, centrifuged, and resuspended. BMSCs were cultured in α-MEM supplemented with 10 % FBS (Gibco) and 1 % Penicillin-Streptomycin (Gibco) at 37 °C in a humidified atmosphere containing 5 % CO_2_. The medium was replaced every two days. Cells between passages 3 and 5 were used for all experiments.

#### 2.6.2 Cytocompatibility test

Scaffold discs (Ø 10 mm × 1 mm) were sterilized by ethanol washing and UV irradiation, and then placed in 48-well plates. BMSCs were inoculated onto the scaffolds using a dynamic seeding method. For this, pre-treated scaffolds were placed in 50 mL centrifuge tubes. A 12 mL cell suspension (2 × 10^5^ cells/mL) was added to each tube. Dynamic inoculation was then performed on a rotary mixer at 60 rpm for 2 h. After 2 h, the scaffolds were carefully removed from the centrifuge tubes and transferred to 24-well plates. Subsequently, the plates containing the scaffolds were pre-incubated at 37 °C in a 5 % CO_2_ incubator for 60 min. Then, 1 mL of α-MEM culture medium supplemented with 10 % fetal bovine serum was added to each well for *in vitro* co-culture. The cell seeding protocol detailed above was utilized for all subsequent cellular experiments.

Cell viability and proliferation were assessed at 1, 4, and 7 days using the Cell Counting Kit-8 (CCK-8; NCM Biotech, Suzhou, China). Briefly, for the CCK-8 assay, scaffolds were transferred to new wells. CCK-8 working solution was added, and the scaffolds were incubated for 2 h at 37 °C. The absorbance of the supernatant was then measured at 450 nm using a microplate reader (BioTek Synergy H1, Winooski, USA). Cell viability at day 4 was also visualized using a Live/Dead Cell Imaging Kit (Proteintech, Wuhan, China). Cells were stained with Calcein-AM (live cells, green) and Propidium Iodide (dead cells, red) and then observed under a laser scanning confocal microscope (LSCM; Leica SP8, Wetzlar, Germany). Cell viability percentage was quantified from images using ImageJ software.

For morphological observation, BMSCs cultured on scaffolds for 1 day were fixed overnight with 4 % paraformaldehyde (PFA). The samples were then dehydrated through a graded ethanol series (50 %, 70 %, 90 %, and 100 %). Subsequently, they were either critical-point dried or air-dried, sputter-coated with gold, and observed by SEM.

#### 2.6.3 Alkaline phosphatase (ALP) activity quantification and staining

For ALP activity quantification, BMSCs were dynamically inoculated onto scaffolds. After 7 and 14 days of incubation, the scaffolds were transferred to new well plates. Cells were then lysed using 0.1 % Triton X-100 lysing solution and subjected to three freeze-thaw cycles (−20 °C and 37 °C). The ALP activity in the lysates was quantitatively analyzed using an ALP Assay Kit (Beyotime, Shanghai, China) according to the manufacturer's instructions. Optical density (OD) values were recorded at 405 nm. ALP activity was normalized to the total protein content of each sample.

For qualitative ALP staining, BMSCs were co-cultured with the scaffolds for 7 and 14 days. After the incubation period, the cells were fixed with 4 % paraformaldehyde (PFA) solution and washed three times with PBS. ALP staining was then performed using a BCIP/NBT ALP Staining Kit (Beyotime, Shanghai, China) following the manufacturer's protocol. Stained cells were imaged using a digital camera attached to a microscope for histological evaluation.

#### 2.6.4 Assessment of matrix mineralization

Matrix mineralization was assessed after 21 days of culture in osteogenic medium. Cells were fixed with 4 % PFA and then stained with 2 % Alizarin Red S (ARS) solution (pH 4.2; OriCell, Guangzhou, China) for 30 min at room temperature. After washing with distilled water, stained mineralized nodules were imaged using a microscope. For quantification, the ARS stain was eluted by incubating the scaffolds with 10 % (w/v) cetylpyridinium chloride solution (Solarbio, Beijing, China) for 20 min. The absorbance of the eluate was then measured at 540 nm.

In addition to ARS staining for total mineralization, tetracycline staining was performed to visualize newly deposited mineral. For tetracycline staining, BMSCs were prepared and cultured on scaffolds for 21 days in osteogenic medium as described for ARS staining. After 21 days of culture in osteogenic medium, the scaffolds were labeled by incubation with 5 μg/L tetracycline hydrochloride working solution (Solarbio, Beijing, China) for 60 min at 37 °C. Subsequently, the tetracycline working solution was removed. The scaffolds were then incubated with complete DMEM (containing 10 % FBS) for an additional 60 min at 37 °C and 5 % CO_2_. After this incubation, the scaffolds were gently rinsed twice with PBS. Finally, the cells on the scaffolds were fixed with 4 % paraformaldehyde for 30 min. Images were then acquired using a laser scanning confocal microscope (LSCM; Leica SP8, Wetzlar, Germany) at excitation/emission wavelengths suitable for tetracycline.

#### 2.6.5 Quantitative PCR detection of osteogenic genes

Osteogenic gene expression was assessed by real-time polymerase chain reaction (qRT-PCR). The expression levels of the following key osteogenic markers were evaluated: osteocalcin (*OCN*), runt-related transcription factor-2 (*Runx-2*), bone sialoprotein (*BSP*), osteopontin (*OPN*), collagen type I (*Col-Ⅰ*), and osterix (*Osx*). BMSCs were dynamically inoculated onto composite porous scaffolds. Total RNA was then extracted from the cells using an RNA extraction kit (Mei5 Biotechnology Co., Ltd., Beijing, China). RNA samples were reverse-transcribed into cDNA using an M5 Super plus qPCR RT kit with gDNA remover (Mei5 Biotechnology Co., Ltd., Beijing, China). Real-time quantitative PCR was subsequently performed using ChamQ SYBR qPCR Master Mix (Vazyme, Nanjing, China) on a QuantGene 9600 real-time PCR system (Bioer Technology, Hangzhou, China). GAPDH was used as the reference gene for normalization. Primer sequences are listed in Table 2. Relative gene expression was calculated using the 2^−^ΔΔCt method.

**Table 2:** Primer pairs used to measure osteogenesis-related genes in qPCR.

Gene	Forward primer sequence (5‘-3’)	Reverse primer sequence (5‘-3’)
*OCN*	GCCCTGACTGCATTCTGCCTCT	TCACCACCTTACTGCCCTCCTG
*Runx-2*	CTTCGTCAGCGTCCTATCAGTTCC	TCCATCAGCGTCAACACCATCATTC
*BSP*	AGCGGAGGAGACAACGGAGAAG	GTCGTGGTGCCATAACTGGTCAG
*OPN*	GACGATGATGACGACGACGATGAC	GTGTGCTGGCAGTGAAGGACTC
*Col-Ⅰ*	TGTTGGTCCTGCTGGCAAGAATG	GTCACCTTGTTCGCCTGTCTCAC
*Osterix*	GCCTACTTACCCGTCTGACTTTGC	CCCTCCAGTTGCCCACTATTGC
*GAPDH*	CCCATTCTTCCACCTTTGAT	CAACTGAGGGCCTCTCTCTT

#### 2.6.6 Western Bolt analysis

For Western Blot analysis, BMSCs were dynamically inoculated onto composite porous scaffolds and cultured for 21 days. After culture, the scaffolds were transferred to a new well plate and clipped into smaller pieces. A mixture of protease inhibitor cocktail (Solarbio, Beijing, China) and protein phosphatase inhibitor cocktail (Solarbio, Beijing, China) was added to RIPA lysis buffer. Cells were then lysed on ice for 30 min to extract total protein. The extracted proteins were denatured by heating at 95 °C for 10 min in SDS-PAGE sample loading buffer (Proteintech, Wuhan, China). Total proteins were separated by sodium dodecyl sulfate-polyacrylamide gel electrophoresis (SDS-PAGE). The separated proteins were then transferred onto a polyvinylidene difluoride (PVDF) membrane (Merck Millipore, Darmstadt, Germany). The membranes were blocked with 5 % (w/v) skimmed milk powder (Yili, Inner Mongolia, China) in TBST for 1 h.

Membranes were incubated overnight at 4 °C with primary antibodies against: Osteopontin (OPN, 1:3000, Proteintech), Bone sialoprotein (BSP, 1:4000, Proteintech), Collagen type I (Col-Ⅰ, 1:3000, Proteintech), and Tubulin (1:4000, Proteintech). After washing three times with TBST, the membranes were incubated with horseradish peroxidase (HRP)-conjugated secondary antibodies (1:5000, Proteintech) for 1 h. Protein bands were visualized using an enhanced chemiluminescence detection kit (zhuangzhibio, Xian, China). Images were captured using a Chemiluminescent Imaging System (Share-Bio, Shanghai, China). The relative intensity of the protein bands was quantified using Image J software.

### 2.7 The effect of PEMF on *in vitro* osteogenesis of composite porous scaffolds

Based on the initial osteogenesis results, the synergistic effect of PEMF was investigated. The study groups included PLA/HA, 4 FeCu-MOF/PLA/HA, and 8 FeCu-MOF/PLA/HA, each with or without PEMF treatment. BMSCs were seeded onto these scaffolds and cultured as previously described. Scaffolds in the PEMF-treated groups (designated as PLA/HA + PEMF, 4 FeCu-MOF/PLA/HA + PEMF, and 8 FeCu-MOF/PLA/HA + PEMF) were exposed daily to PEMF using a PEMF stimulator (GS-100A, Jinan Chuangbo Technology Co., Ltd., Shandong, China). The PEMF parameters were a frequency of 16 Hz, a magnetic intensity of 3.6 mT, and a duration of 60 min per day. These parameters were selected based on our preliminary optimization experiments, which indicated their efficacy in promoting BMSC osteogenic differentiation. Control groups (non-PEMF treated) received identical handling but without active PEMF exposure (sham treatment). Cell proliferation (CCK-8 assay), ALP activity/staining, and mineralization (ARS/tetracycline staining) were assessed at predetermined time points.

### 2.8 *In vivo* animal assessments

#### 2.8.1 *In vivo* compatibility of composite porous scaffolds

All animal experimental procedures were approved by the Institutional Animal Care and Use Committee (IACUC) of Northwest University (No:ACUC2021045) and conducted in accordance with institutional guidelines. Adult male SD rats, weighing 220–250 g, were used for the study. The rats were anesthetized via an intraperitoneal (i.p.) injection of 1 % sodium pentobarbital (50 mg/kg body weight). Scaffold discs (Ø 10 mm × 1 mm), sterilized by ethanol washing and UV irradiation, were implanted subcutaneously into dorsal pockets on the rats. Rats were euthanized at 1 and 4 weeks post-implantation. Surrounding tissues, including the implants, were harvested. The samples were then fixed in 4 % PFA, dehydrated through a graded ethanol series, embedded in paraffin, and sectioned. Sections were subsequently stained with hematoxylin and eosin (H&E) for histological assessment. Immunohistochemical staining for CD31 was also performed on samples collected at 4 weeks post-implantation.

#### 2.8.2 Bone defect model and scaffold implantation

Adult male SD rats (weighing 220–250 g) were anesthetized, and the surgical scalp area was subsequently shaved and sterilized. A full-thickness cranial defect (5 mm diameter) was created in the parietal bone using a dental drill under constant saline irrigation. Rats were randomly assigned to six groups (n = 6 per group): Control (empty defect); PLA/HA control; 4 FeCu-MOF/PLA/HA; 4 FeCu-MOF/PLA/HA + PEMF; 8 FeCu-MOF/PLA/HA; 8 FeCu-MOF/PLA/HA + PEMF. Scaffolds were press-fitted into the defects. For groups receiving PEMF treatment, the entire rat was placed within the PEMF coil for stimulation. The stimulation protocol commenced on the day following surgery to allow for initial recovery. During the treatment sessions, the rats were conscious and allowed to move freely within their custom-made, non-metallic home cages, which were placed at the center of the coil. This standard, unrestrained protocol is designed to minimize stress and does not require the use of anesthesia or analgesia during stimulation. The core PEMF parameters—frequency (16 Hz) and magnetic intensity (3.6 mT)—were consistent with those used in the *in vitro* experiments. However, the total daily treatment duration for *in vivo* studies was 120 min.

#### 2.8.3 Radiographic evaluation

Rats (n = 3 per group per time point) were euthanized at 4 and 8 weeks post-implantation. Cranial specimens encompassing the defect area were harvested and subsequently fixed in 4 % PFA for 48 h. Samples were scanned using a micro-computed tomography (micro-CT) system (model AX-2000; Aoying Detection Technology (Ningbo) Co., Ltd., China). Scanning was performed at a voltage of 80 kVp, a current of 70 μA, and a voxel size of 12.16 × 12.16 × 12.16 μm^3^. 3D images were reconstructed using AYRecon_Auto_V3.5 software. Quantitative analysis was performed using VG Studio MAX 3.5 software within a cylindrical region of interest corresponding to the original 5 mm diameter defect volume. The following parameters were calculated: bone mineral density (BMD), bone volume relative to tissue volume (BV/TV), trabecular number (Tb.N), and trabecular separation (Tb.Sp).

#### 2.8.4 Histological tests and immunohistochemical analysis

After micro-CT scanning, cranial specimens were decalcified in 10 % ethylenediaminetetraacetic acid solution (pH 7.4) for 4 weeks, with the decalcification solution changed every 7 days. Decalcified samples were subsequently dehydrated through a graded ethanol series, cleared with xylene, embedded in paraffin, and sectioned transversely through the center of the defect at a thickness of 5 μm. Sections were stained with H&E to assess general morphology. Masson's trichrome staining was performed to evaluate collagen deposition and bone maturation.

For immunohistochemistry (IHC), sections underwent heat-induced epitope retrieval in citrate buffer. Sections were then incubated overnight at 4 °C with the following primary antibodies: anti-OPN (1:700, Servicebio), anti-Collagen type I (Col-Ⅰ, 1:500, Servicebio), anti-OCN (1:400, Servicebio), and anti-CD31 (1:400, Servicebio). After washing with PBS, sections were incubated with an appropriate horseradish peroxidase (HRP)-conjugated secondary antibody (1:300; Servicebio) for 1 h. The signal was visualized using a DAB (3,3′-diaminobenzidine) substrate kit. Images were captured using a light microscope.

### 2.9 Data statistics

All quantitative data are expressed as mean ± standard error of the mean (SEM). Statistical analyses were performed using GraphPad Prism 9.0 software and Origin 2024. One-way analysis of variance (ANOVA) followed by the Student-Newman-Keuls (SNK) post hoc test was used for multiple group comparisons. A *p*-value <0.05 was considered statistically significant. All experiments were repeated at least 3 times.

## 3 Results

### 3.1 Synthesis and characterization of FeCu-MOF nanoparticles

**Fig. 1:**
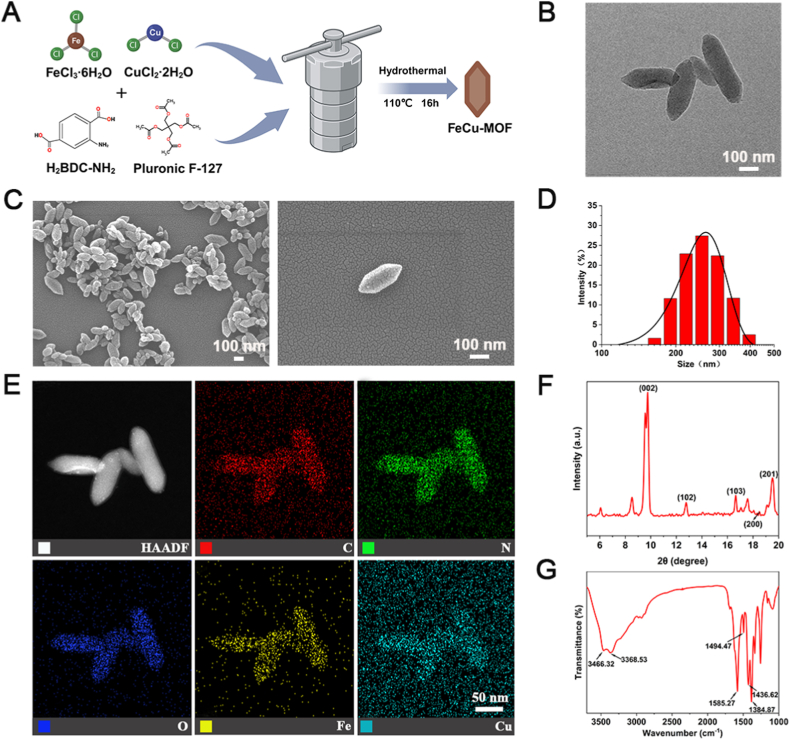
Synthesis and characterization of bimetallic FeCu-MOF nanoparticles. (A) Schematic illustration of the one-pot hydrothermal synthesis process. (B, C) Representative TEM and SEM images, respectively, showing the primary morphology of biconical hexagonal prisms with some heterogeneity. (D) DLS analysis reveals an average hydrodynamic diameter of ∼250 nm in aqueous dispersion. (E) High-angle annular dark-field-STEM imaging with EDS elemental mapping confirms the bimetallic composition, with a homogeneous intra-particle distribution of Fe, Cu, C, N, and O. (F) XRD pattern exhibits a sharp, characteristic peak at 2θ ≈ 9.8°, confirming the crystalline structure of MOF. (G) FTIR spectrum verifies the chemical structure, showing characteristic N-H stretching and carboxylate vibration bands, indicating successful coordination between metal ions and the NH_2_-BDC organic ligand. Scale bars: 100 nm in B and C; 50 nm in E.

Bimetallic FeCu-MOF nanoparticles were designed as carriers for the programmed co-release of Fe^3+^ and Cu^2+^ ions (schematically shown in Fig. 1A). SEM and TEM revealed a well-defined biconical hexagonal prism as the predominant morphology, accompanied by a degree of polymorphism, including smaller ellipsoidal and irregular particles (Fig. 1B and C). This morphological heterogeneity is likely due to subtle variations in local nucleation and growth kinetics during the solvothermal synthesis. However, as the subsequent characterization and biological performance data demonstrate, this variability did not adversely impact the overall functionality of the composite material. This specific morphology and rough surface are expected to influence their interaction with the surrounding matrix and subsequent ion release/exchange. DLS analysis indicated an average hydrodynamic diameter of approximately 250 nm with a narrow size distribution (Fig. 1D). EDS mapping confirmed the presence and homogeneous distribution of Fe, Cu, C, N, and O elements throughout the FeCu-MOF nanoparticles (Fig. 1E), confirming successful and uniform incorporation of both metal ions, essential for their designed co-release.

The crystalline structure was confirmed by XRD (Fig. 1F). The pattern is dominated by a prominent, sharp diffraction peak at 2θ ≈ 9.8°, characteristic of the desired FeCu-MOF framework and indicative of high overall crystallinity. Minor, broader peaks are also present, likely corresponding to a small fraction of an amorphous phase or trace unreacted starting materials. FTIR verified the coordination structure (Fig. 1G), with characteristic peaks for N-H stretching (3466 cm^−1^, 3369 cm^−1^) and carboxylate symmetric/asymmetric vibrations (1384–1585 cm^−1^), indicating coordination with metal ions in a bidentate bridging mode. This specific coordination geometry is crucial for stable metal ion incorporation and dictates their subsequent release characteristics. Collectively, these characterizations confirm the successful synthesis of crystalline, bimetallic FeCu-MOF nanoparticles possessing the desired morphology, composition, and key structural features. These attributes provide the necessary structural foundation for their intended role as a precisely controlled ion-releasing component.

### 3.2 Characterization of composite porous scaffolds

**Fig. 2:**
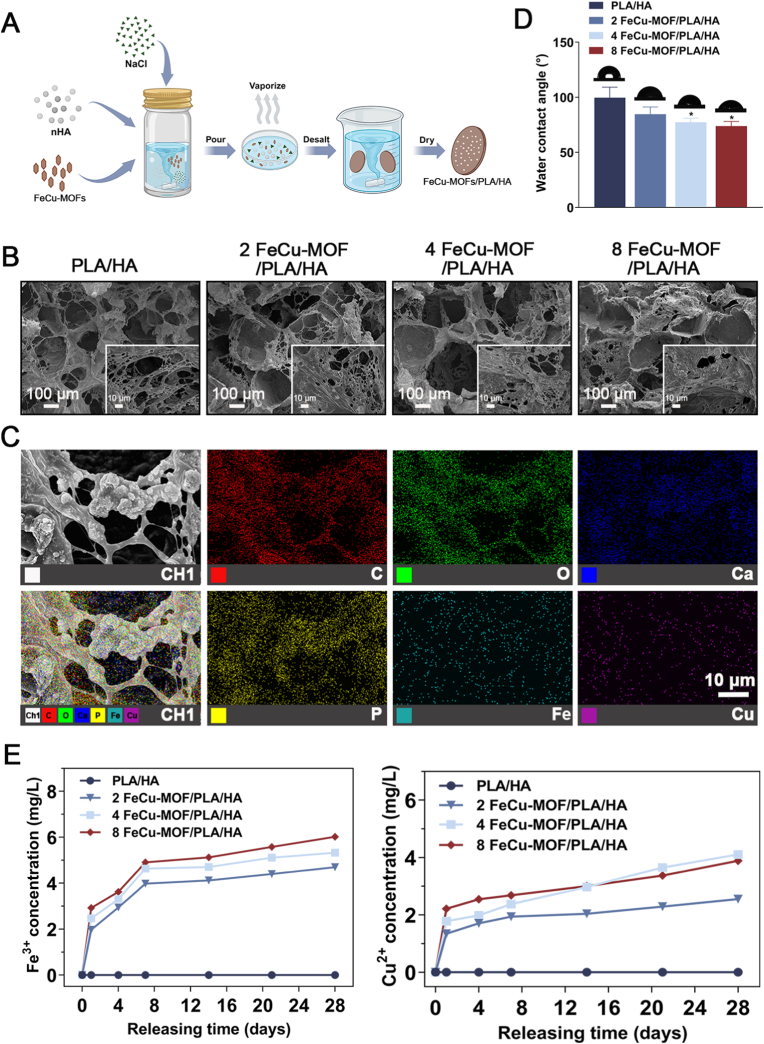
Fabrication and physicochemical characterization of FeCu-MOF/PLA/HA composite scaffolds. (A) Schematic of the fabrication process using solvent casting and particulate leaching to create porous scaffolds. (B) SEM images of scaffold cross-sections. Low-magnification images show a hierarchical, interconnected porous architecture across all groups. High-magnification insets highlight the increased nanoscale surface roughness of pore walls in FeCu-MOF scaffolds compared to the smoother PLA/HA control. (C) EDS elemental mapping of the 4 % FeCu-MOF/PLA/HA scaffold, confirming the uniform distribution of Fe and Cu throughout the matrix from the incorporated FeCu-MOF. (D) Static water contact angle measurements reveal enhanced hydrophilicity with increasing FeCu-MOF content. (E) Cumulative release profiles of Fe^3+^ and Cu^2+^ from the scaffolds over 28 days in PBS, showing sustained, dose-dependent co-release of both ions from MOF-containing scaffolds. ∗*p* < 0.05 indicates significant difference vs. PLA/HA control at Day 28. Scale bars: 100 μm in B; 10 μm for the insets in B and for C.

Composite porous scaffolds of FeCu-MOF/PLA/HA were fabricated by incorporating HA and varying concentrations of FeCu-MOF into a PLA matrix using a solvent casting-particulate leaching technique (Fig. 2A). Macroscopically, the scaffold color transitioned from white (PLA/HA) to brown with increasing FeCu-MOF content (2 %, 4 %, and 8 % wt/wt). SEM analysis revealed that all scaffold groups possessed a hierarchically porous architecture (Fig. 2B) with irregular polygonal macropores (200–400 μm) interconnected by smaller micropores or windows (100–200 μm). This hierarchical porous structure is designed to facilitate cell infiltration, nutrient/waste transport, and subsequent tissue ingrowth, mimicking key architectural features of natural cancellous bone. Incorporating FeCu-MOF visibly increased the surface roughness of the pore walls compared to the PLA/HA control group, a modification expected to directly enhance subsequent cell attachment and spreading (Fig. 2B, insets). To confirm nanoparticle distribution, SEM-EDS elemental mapping was performed on the 4 % FeCu-MOF scaffold's cross-section. The results revealed a uniform distribution of Fe and Cu elements within the PLA/HA matrix, indicating homogeneous dispersion of FeCu-MOF nanoparticles without significant agglomeration (Fig. 2C). This even distribution is crucial for achieving predictable biological outcomes.

Surface hydrophilicity was evaluated by water contact angle measurements (Fig. 2D). The pristine PLA/HA scaffold exhibited hydrophobic behavior (95.5 ± 2.5°). FeCu-MOF incorporation progressively decreased the water contact angle: 83.1 ± 4.1° (2 %), 76.5 ± 2.4° (4 %), and 67.3 ± 5.0° (8 % FeCu-MOF). This enhancement in surface wettability is desirable as it generally promotes protein adsorption and cell adhesion, crucial initial steps for tissue integration.

The programmed co-release of Fe^3+^ and Cu^2+^ ions was quantified by ICP-MS in PBS over 28 days (Fig. 2E). All FeCu-MOF-containing scaffolds exhibited sustained ion release. Distinct release kinetics were observed for the two metal ions. Fe^3+^ demonstrated an initial, relatively faster release phase within the first 7 days, which was then followed by a more gradual and sustained liberation. In contrast, Cu^2+^ release was characterized by a slower and consistently gradual profile throughout the entire 28-day period, without an initial burst. At day 28, cumulative release from the 8 FeCu-MOF/PLA/HA group reached approximately 6.01 mg/L for Fe^3+^ and 3.98 mg/L for Cu^2+^. As anticipated, the total amounts of ions released were dependent on the initial FeCu-MOF loading concentration within the scaffolds.

### 3.3 *In vitro* biocompatibility of composite porous scaffolds

**Fig. 3:**
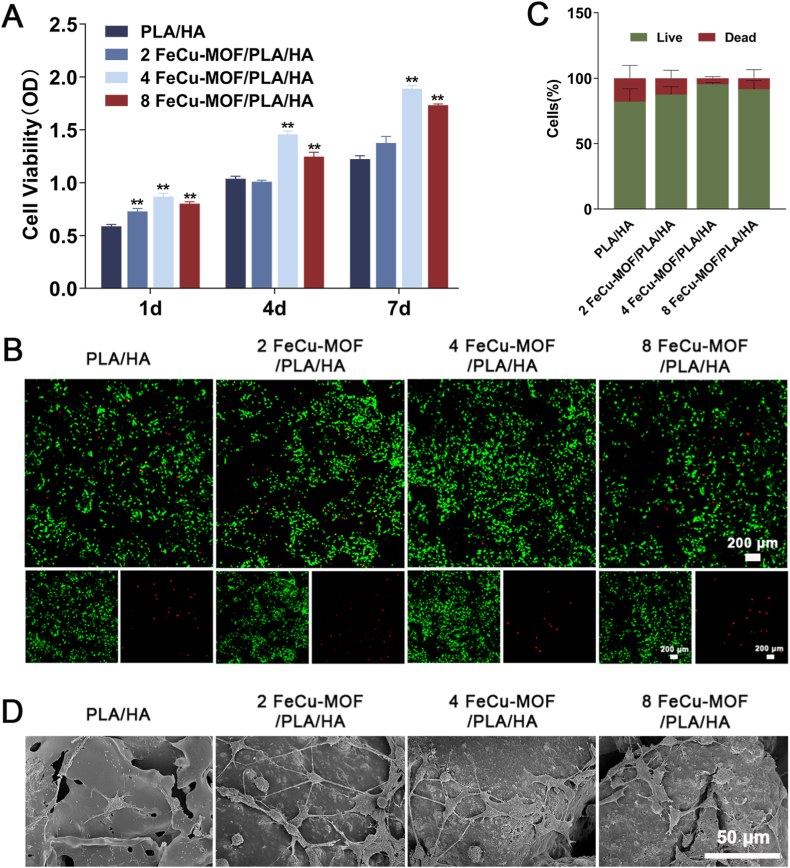
*In vitro* Biocompatibility of FeCu-MOF/PLA/HA Composite Scaffolds with BMSCs. (A) Time-dependent proliferation of BMSCs over 7 days, assessed by CCK-8 assay, showing significantly enhanced proliferation on 4 % and 8 % FeCu-MOF scaffolds. (B, C) Cell viability after 4 days of culture. (B) Live/Dead fluorescence microscopy images show a high proportion of viable cells (green, Calcein-AM) and very few dead cells (red, Propidium Iodide) across all groups. (C) Quantitative analysis of fluorescence images confirms excellent cell viability (>95 %) for all scaffold formulations. (D) SEM images after 1 day of culture reveal robust BMSCs adhesion and spreading. Cells on 4 % FeCu-MOF scaffolds exhibit well-spread morphology with extensive filopodia. ∗*p* < 0.05, ∗∗*p* < 0.01 compared to PLA/HA control at corresponding time points. Scale bars: 200 μm in B; 50 μm in D. (For interpretation of the references to color in this figure legend, the reader is referred to the Web version of this article.)

Cytocompatibility was evaluated using rat BMSCs. Cell proliferation by CCK-8 assay showed a time-dependent increase on all groups over 7 days (Fig. 3A). At days 4 and 7, BMSCs on 4 % and 8 % FeCu-MOF scaffolds showed significantly higher viability (*p* < 0.01) than the PLA/HA control. The 4 % FeCu-MOF group consistently supported the highest proliferation at these time points.

Live/Dead staining at day 4 supported proliferation findings (Fig. 3B). All scaffolds showed predominant green fluorescence, indicating excellent biocompatibility. The 4 FeCu-MOF/PLA/HA group appeared to support the highest density of viable cells. Quantitative analysis confirmed high live cell percentage (>95 %) across all groups (Fig. 3C).

SEM observation after 1 day showed good cell adhesion and spreading on all scaffold surfaces (Fig. 3D), with cells displaying spindle-shaped morphology and extended filopodia. Cells on the 4 FeCu-MOF/PLA/HA scaffold appeared particularly well-spread with numerous lamellipodia, suggesting enhanced cell-surface interaction. While direct intercellular connections were not quantified, qualitative observation suggested robust cell growth. These results collectively demonstrate that the FeCu-MOF/PLA/HA scaffolds support BMSCs adhesion, proliferation, and viability. The 4 % FeCu-MOF concentration consistently exhibited favorable characteristics for these cellular responses, likely due to a combination of its optimized surface properties and a beneficial ion release profile.

### 3.4 *In vitro* osteogenic differentiation potential

**Fig. 4:**
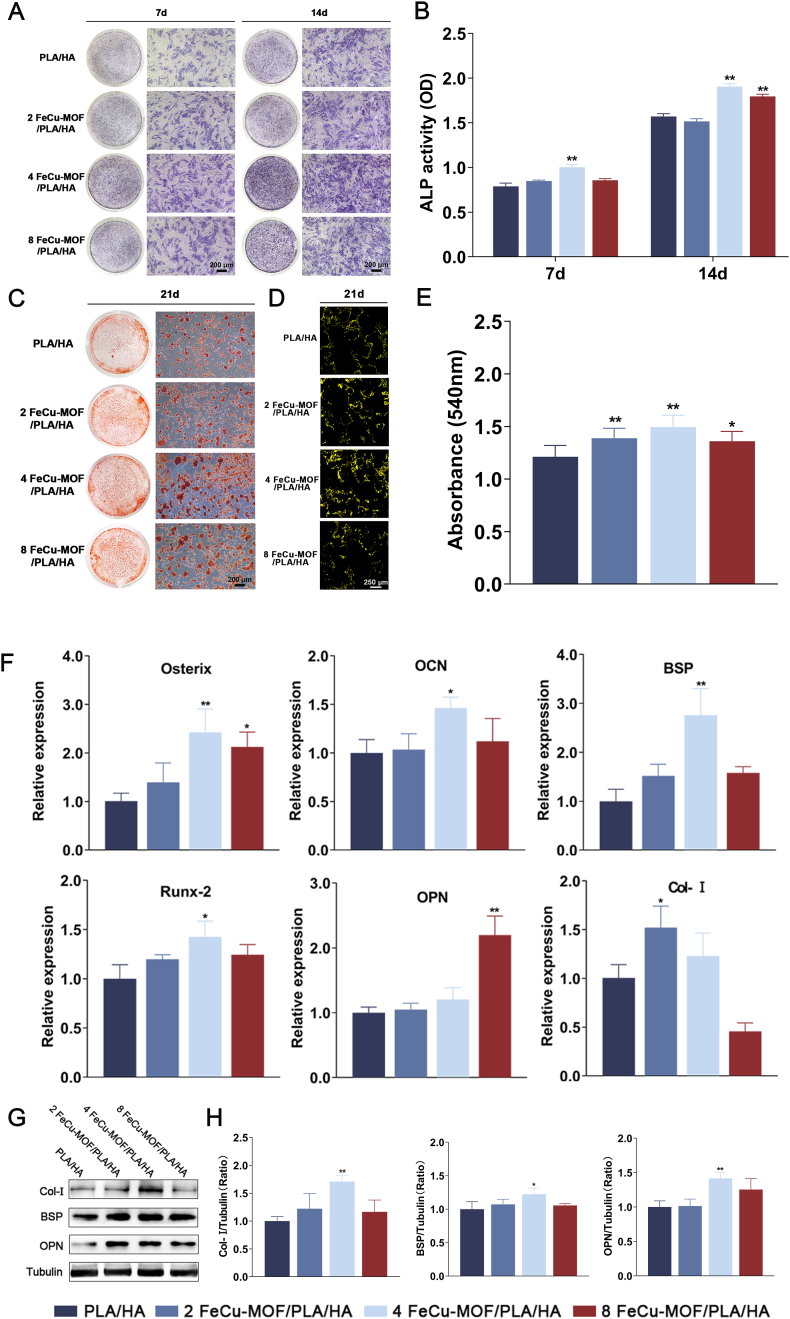
Osteoinductive properties of the composite scaffolds demonstrated by *in vitro* differentiation of BMSCs. (A, B) Early osteogenic differentiation assessed by ALP activity. (A) Qualitative ALP staining at days 7 and 14 reveals enhanced activity in all FeCu-MOF groups. (B) Quantitative analysis confirms this trend, with the 4 % FeCu-MOF group showing the highest ALP activity. (C, D, E) Late-stage osteogenic mineralization at day 21. (C) ARS staining and (D) Tetracycline hydrochloride fluorescence staining both demonstrate significantly increased mineral deposition in FeCu-MOF groups, particularly in the 4 % group. (E) Quantitative of eluted ARS stain supports these findings, showing the highest mineralization in the 4 % group. (F) Osteogenesis-related gene expression at day 21. Quantitative RT-PCR shows significant upregulation of key osteogenic markers (*Osterix*, *OCN*, *BSP*, *Runx-2*) in the 4 % FeCu-MOF group. (G, H) Osteogenesis-related protein expression at day 21. (G) Western blot analysis and (H) Densitometric quantification confirm increased protein levels of Col-I, BSP, and OPN, with the highest expression in the 4 % FeCu-MOF group. α-Tubulin served as the loading control. ∗*p* < 0.05, ∗∗*p* < 0.01 compared to the PLA/HA control group. Scale bar: 200 μm in A and C; 250 μm in D.

The intrinsic osteoinductive properties of the scaffolds were assessed by evaluating ALP activity, matrix mineralization, and osteogenic gene/protein expression in BMSCs cultured without PEMF stimulation. ALP staining at days 7 and 14 showed time-dependent increases in intensity across all groups (Fig. 4A). All FeCu-MOF-containing groups exhibited enhanced ALP staining intensity compared to the PLA/HA control. The 4 FeCu-MOF/PLA/HA group displayed the most intense staining. Quantitative analysis confirmed significantly higher ALP levels (*p* < 0.01) in all FeCu-MOF groups compared to the PLA/HA control at both day 7 and 14, with the 4 % group showing the highest activity (Fig. 4B). This suggests that the FeCu-MOF component promoted early osteogenic differentiation, likely through the release of bioactive ions.

Matrix mineralization, a marker of late-stage osteogenesis, was evaluated by ARS staining after 21 days (Fig. 4C). All FeCu-MOF-containing groups showed markedly increased formation of calcified nodules compared to the PLA/HA control group. The 4 FeCu-MOF/PLA/HA group exhibited the most extensive mineralization. Tetracycline hydrochloride staining at day 21 further corroborated these findings (Fig. 4D). Quantitative analysis of the eluted ARS stain confirmed significantly higher mineral deposition (*p* < 0.01) in the 4 % FeCu-MOF group compared to all other groups (Fig. 4E). These results underscore the potent ability of the FeCu-MOF scaffolds, likely via ion release, to drive the mineralization process.

Expression levels of key osteogenic genes were measured by qRT-PCR after 21 days (Fig. 4F). Compared to the PLA/HA control, the 4 FeCu-MOF/PLA/HA group exhibited significantly upregulated expression of crucial transcription factors (*Runx-2*, *Osterix*) and late osteogenic markers (*OCN*, *BSP*) (*p* < 0.05 or *p* < 0.01). The 2 % and 8 % FeCu-MOF groups also showed a trend of elevated expression. Expression of *OPN* and *Col-Ⅰ* also showed increased levels in the FeCu-MOF-containing groups, with the 4 % group exhibiting the most pronounced trend. However, these differences relative to the PLA/HA control were not statistically significant (*p* > 0.05) for these specific markers at this time point.

Western blot analysis assessed protein expression levels of Col-Ⅰ, BSP, and OPN after 21 days (Fig. 4G). Consistent with the gene expression data, all FeCu-MOF groups showed higher levels of these osteogenic proteins compared to the PLA/HA control. Quantitative analysis revealed significantly higher expression of Col-Ⅰ, BSP, and OPN (*p* < 0.01 or *p* < 0.05) in the 4 FeCu-MOF/PLA/HA group relative to the PLA/HA control (Fig. 4H). Taken together, these comprehensive *in vitro* results demonstrate that the FeCu-MOF/PLA/HA scaffolds possess significant intrinsic osteoinductive properties. Among the tested formulations, the 4 % FeCu-MOF concentration consistently yielded the most potent osteogenic response. This superior performance is likely attributed to the release of bioactive Fe^3+^ and Cu^2+^ ions at this specific loading, effectively promoting BMSC differentiation and matrix mineralization.

### 3.5 Synergistic effect of PEMF on *in vitro* osteogenesis

**Fig. 5:**
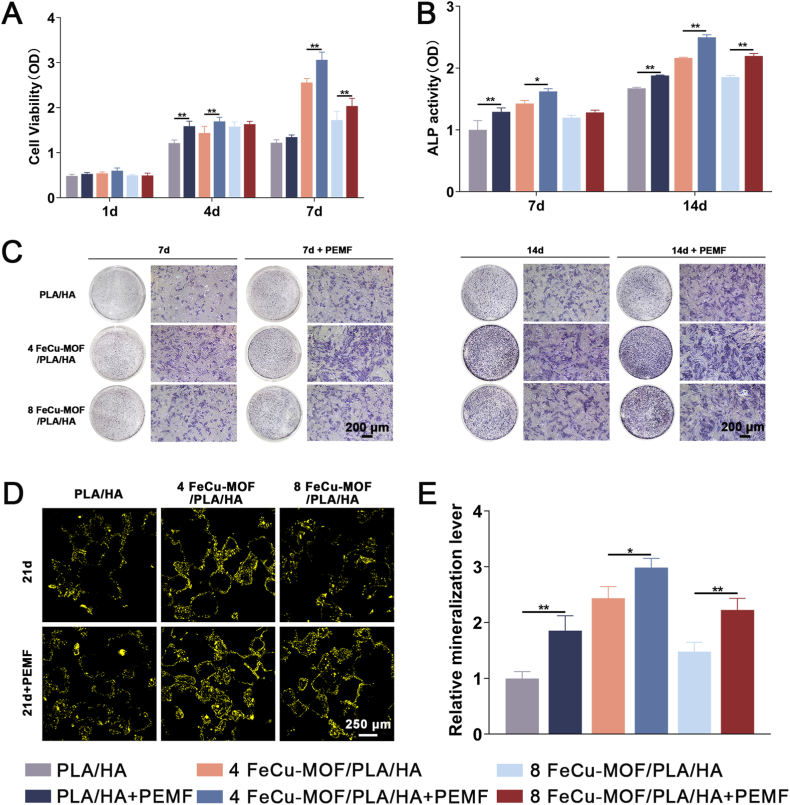
Synergistic effect of PEMF stimulation on the osteoinductive properties of FeCu-MOF/PLA/HA scaffolds. BMSCs were cultured on PLA/HA, 4 % FeCu-MOF/PLA/HA, and 8 % FeCu-MOF/PLA/HA scaffolds with or without daily PEMF treatment. (A) Cell proliferation over 7 days. (B, C) Early osteogenic differentiation at days 7 and 14. (C) Representative ALP staining images and (B) Quantitative analysis reveal that PEMF markedly amplified ALP activity, with the 4 % FeCu-MOF + PEMF group showing the highest levels. (D, E) Late-stage matrix mineralization at day 21. (D) Representative tetracycline hydrochloride fluorescence staining and (E) Quantitative analysis demonstrate that PEMF synergistically enhanced mineral deposition, with the 4 % FeCu-MOF + PEMF group exhibiting the highest mineralization. ∗*p* < 0.05, ∗∗*p* < 0.01, indicating significant differences between PEMF-treated groups and their corresponding non-PEMF counterparts. Scale bar: 200 μm in C; 250 μm in D.

To investigate the hypothesized synergistic effect of PEMF stimulation with the ion-releasing scaffolds, selected groups (PLA/HA, 4 FeCu-MOF/PLA/HA, and 8 FeCu-MOF/PLA/HA) were subjected to daily PEMF stimulation. Cell proliferation by CCK-8 assay showed a time-dependent increase on all groups over 7 days (Fig. 5A). A clear synergistic effect of PEMF emerged from day 4 onwards. To parse the standalone contribution of the physical stimulus, we first compared the PLA/HA + PEMF group to the PLA/HA control. This comparison revealed that PEMF treatment alone moderately but significantly enhanced proliferation, confirming its intrinsic bio-stimulatory effect (*p* < 0.05). However, PEMF treatment significantly enhanced proliferation in both PLA/HA + PEMF and 4 % FeCu-MOF + PEMF groups compared to their non-PEMF counterparts (*p* < 0.01). The 4 FeCu-MOF/PLA/HA + PEMF group exhibited the highest proliferation rate among all conditions at days 4 and 7 (*p* < 0.01 compared to other groups).

The synergistic effect of PEMF on osteogenic differentiation was further evaluated by assessing ALP activity and mineralization. ALP staining at days 7 and 14 revealed enhanced intensity in all PEMF-treated groups compared to their respective non-PEMF controls (Fig. 5C). Quantitative ALP activity assays confirmed significantly higher levels in PEMF-treated groups at both time points (*p* < 0.01 or *p* < 0.05) (Fig. 5B). The 4 FeCu-MOF/PLA/HA + PEMF group showed the highest ALP activity. Mineralization by tetracycline hydrochloride staining after 21 days also demonstrated synergistic enhancement by PEMF (Fig. 5D). Quantitative analysis confirmed significantly increased mineralization levels in all PEMF groups (*p* < 0.01 or *p* < 0.05) (Fig. 5E). The 4 FeCu-MOF/PLA/HA + PEMF group achieved the highest level of mineralization.

These results demonstrate a clear synergistic effect induced by daily PEMF stimulation. Specifically, PEMF treatment of BMSCs cultured on both PLA/HA and FeCu-MOF-containing scaffolds significantly enhanced cell proliferation, ALP activity, and matrix mineralization. These outcomes were markedly improved compared to those observed with scaffolds lacking PEMF treatment. The 4 FeCu-MOF/PLA/HA + PEMF group consistently demonstrated the most significant osteoinductive responses under these combined chemophysical conditions.

### 3.6 *In vivo* bone regeneration assessment

#### 3.6.1 *In vivo* biocompatibility and angiogenesis

**Fig. 6:**
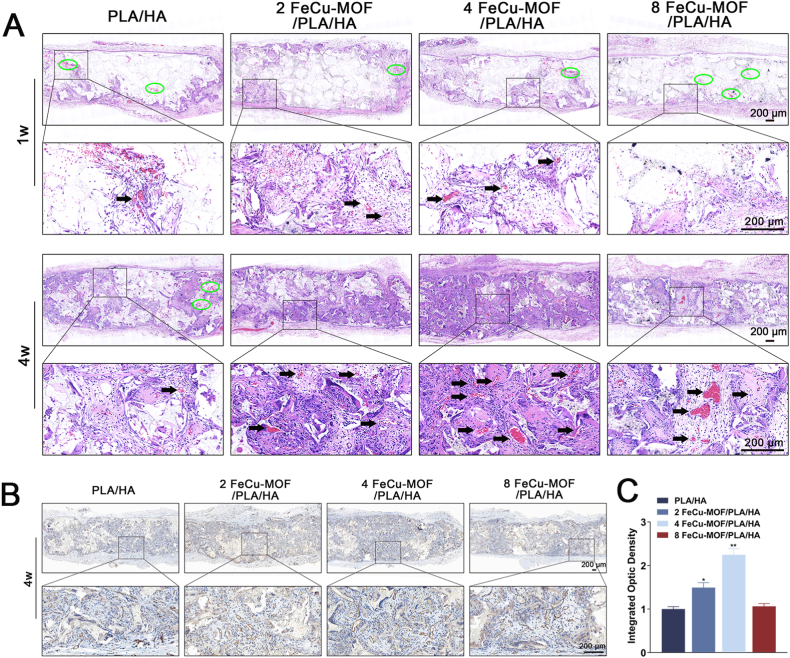
*In vivo* biocompatibility and pro-angiogenic activity of the scaffolds after subcutaneous implantation in rats. (A) Representative H&E stained sections of tissues surrounding the implants at 1 and 4 weeks. All scaffold types elicited minimal inflammatory responses (green circles), demonstrating excellent biocompatibility. Progressive tissue infiltration and early vascularization were observed, with black arrows indicating erythrocytes within newly formed blood vessels. (B, C) Assessment of angiogenesis at 4 weeks post-implantation. (B) Immunohistochemical staining for the endothelial marker CD31 (brown), highlighting newly formed blood vessel. (C) Quantitative analysis of CD31-positive area fraction shows that FeCu-MOF scaffolds significantly promoted angiogenesis, with the 4 % FeCu-MOF group exhibiting the strongest effect. ∗*p* < 0.05, ∗∗*p* < 0.01 compared to the PLA/HA control group. Scale bar: 200 μm. (For interpretation of the references to color in this figure legend, the reader is referred to the Web version of this article.)

The *in vivo* tissue response to the scaffolds following subcutaneous implantation was evaluated to assess their biocompatibility and initial tissue integration. H&E staining of tissues harvested at 1 and 4 weeks post-implantation revealed minimal inflammatory responses surrounding all scaffold types, indicating good tissue biocompatibility (Fig. 6A). Gradual scaffold degradation and progressive tissue infiltration were observed over time. At 4 weeks, significant tissue ingrowth was evident, appearing particularly prominent within the 4 FeCu-MOF/PLA/HA scaffold. This group also exhibited some erythrocyte aggregates within the newly formed tissue, suggestive of early vascularization.

To assess their pro-angiogenic potential, IHC staining for the endothelial cell marker CD31 was performed on tissue samples from the scaffolds at 4 weeks post-implantation (Fig. 6B). Positive CD31 staining, indicating blood vessel formation, was observed around and within all scaffold types. The density of CD31-positive vessels appeared highest in the 4 FeCu-MOF/PLA/HA group. Quantitative analysis confirmed significantly higher CD31 expression in the 4 % (*p* < 0.01) and 2 % (*p* < 0.05) FeCu-MOF groups compared to the PLA/HA control (Fig. 6C). The 8 % FeCu-MOF group also showed an increasing trend in CD31 expression compared to the PLA/HA control, although this difference did not reach statistical significance (*p* > 0.05). These findings suggest that the FeCu-MOF component endows the PLA/HA scaffolds with intrinsic pro-angiogenic properties *in vivo*, likely through the controlled release of Cu^2+^ ions.

#### 3.6.2 *In vivo* bone regeneration capacity

**Fig. 7:**
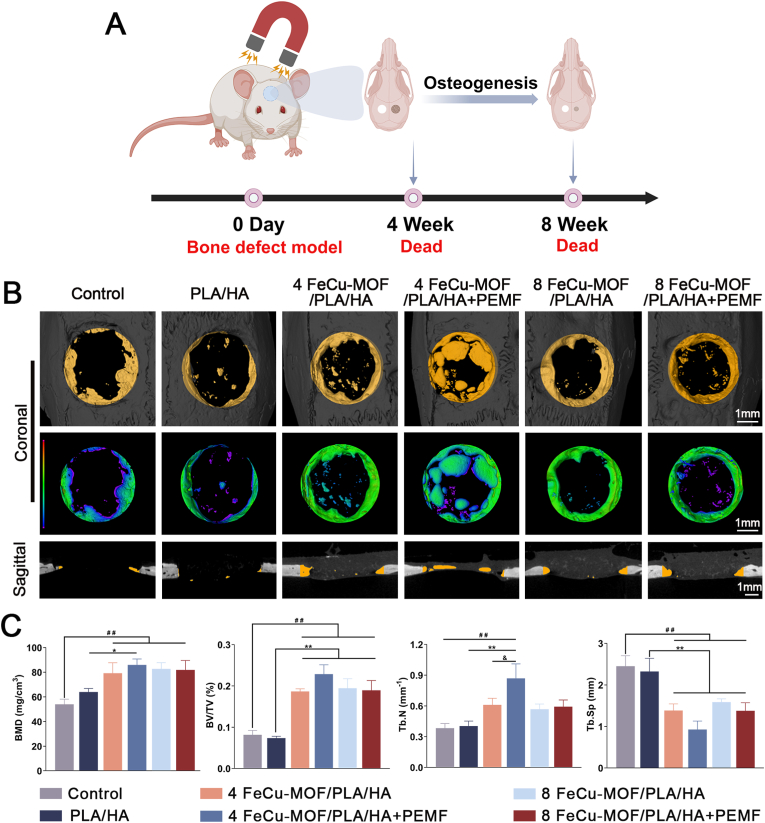
Evaluation of *in vivo* bone regeneration in rat cranial defect model at 8 weeks post-implantation. (A) Schematic overview of the experimental design, showing scaffold implantation and daily PEMF treatment. (B) Representative micro-CT reconstructions of defect sites, including 3D views and coronal/sagittal sections. The color map corresponds to mineral density, ranging from low (blue) to high (red), and visually highlights superior bone formation in FeCu-MOF groups, with the most robust healing observed in PEMF-treated groups. (C) Quantitative analysis of bone regeneration parameters within the region of interest: BMD, BV/TV, Tb.N, and Tb.Sp. Results confirm that FeCu-MOF scaffolds significantly enhanced bone regeneration, with PEMF stimulation further amplifying this effect. Statistical significance: ^##^*p* < 0.01 compared with the empty Control group; ∗*p* < 0.05, ∗∗*p* < 0.01 compared with the PLA/HA group; ^&^*p* < 0.05 indicates a significant synergistic effect when comparing a PEMF-treated group to its corresponding non-PEMF counterpart. (For interpretation of the references to color in this figure legend, the reader is referred to the Web version of this article.)

The bone regeneration efficacy of the engineered chemophysical system was evaluated over an 8-week period in a rat cranial defect model (Fig. 7A). Micro-CT reconstructions at 8 weeks showed varying degrees of defect healing across the experimental groups (Fig. 7B). While the Control and PLA/HA groups displayed minimal new bone formation, substantial new bone formation that bridged the defect was observed in FeCu-MOF-containing scaffolds. Enhanced regeneration was also apparent in all groups receiving PEMF treatment. Visually, the most extensive bone regeneration appeared in the combination groups (4 % and 8 % FeCu-MOF + PEMF), providing initial qualitative evidence of a synergistic chemophysical effect.

Quantitative micro-CT analysis confirmed these observations (Fig. 7C). Compared to the Control and PLA/HA groups, all FeCu-MOF-containing groups (with or without PEMF) demonstrated significantly higher BMD and BV/TV (*p* < 0.01). Tb.N was also significantly increased, while Tb.Sp was generally decreased in these groups. Evidence for a synergistic chemophysical interaction emerged when comparing PEMF-treated groups to their non-PEMF counterparts. PEMF stimulation significantly enhanced Tb.N for the 4 % FeCu-MOF group (*p* < 0.05, marked as & in Fig. 7C). Furthermore, PEMF treatment promoted strong trends towards increased BMD and BV/TV in both the 4 % and 8 % FeCu-MOF groups. These upward trends were evident when these groups were compared to their non-PEMF counterparts. However, it should be noted that these trends did not achieve statistical significance (*p* > 0.05) in the current analysis. Collectively, these quantitative data support the capacity of the FeCu-10.13039/501100005045MOF scaffolds to promote *in vivo* bone regeneration and indicate a clear additive or synergistic benefit when combined with 10.13039/100004049PEMF treatment.

**Fig. 8:**
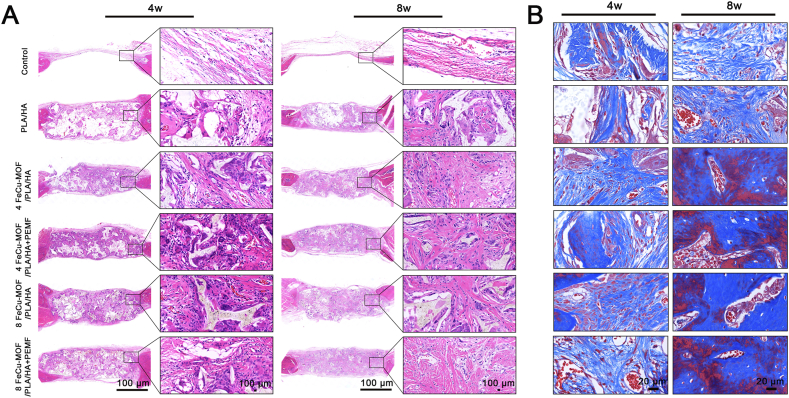
Histological analysis of new bone formation and tissue maturation within the cranial defect at 4 and 8 weeks. (A) H&E staining. The FeCu-MOF groups exhibit significantly more new bone formation, characterized by an organized tissue structure and embedded osteocyte-like cells, compared to controls. PEMF stimulation appears to accelerate tissue integration and maturation. Black squares indicate regions shown at higher magnification on the right. (B) Masson's trichrome staining. Collagen (blue) and mineralized bone (red/pink) are visualized, showing that at 8 weeks, the 4 % and 8 % FeCu-MOF + PEMF groups display the most mature and well-integrated bone tissue. This is evidenced by extensive areas of red-stained mineralized matrix seamlessly connected to the underlying host bone. Scale bars: 100 μm in A; 20 μm in B. (For interpretation of the references to color in this figure legend, the reader is referred to the Web version of this article.)

Histological analysis using H&E and Masson's trichrome staining provided further evidence at 4 and 8 weeks (Fig. 8A and B). Minimal inflammation was observed in all implanted groups. Compared to the Control and PLA/HA groups, the FeCu-MOF-containing groups exhibited significantly more new bone tissue formation, characterized by organized collagen fibers and osteocyte-like cells. PEMF treatment appeared to accelerate scaffold degradation and promote denser collagen alignment. At 8 weeks, the 4 FeCu-MOF/PLA/HA + PEMF group displayed the most mature and well-integrated bone tissue. This newly formed tissue was seamlessly connected with the underlying host bone and was notably permeated by abundant vascular structures within the regenerated area. These histological features strongly indicated successful vascularized osteogenesis, driven by the inherent chemophysical synergy of the treatment.

**Fig. 9:**
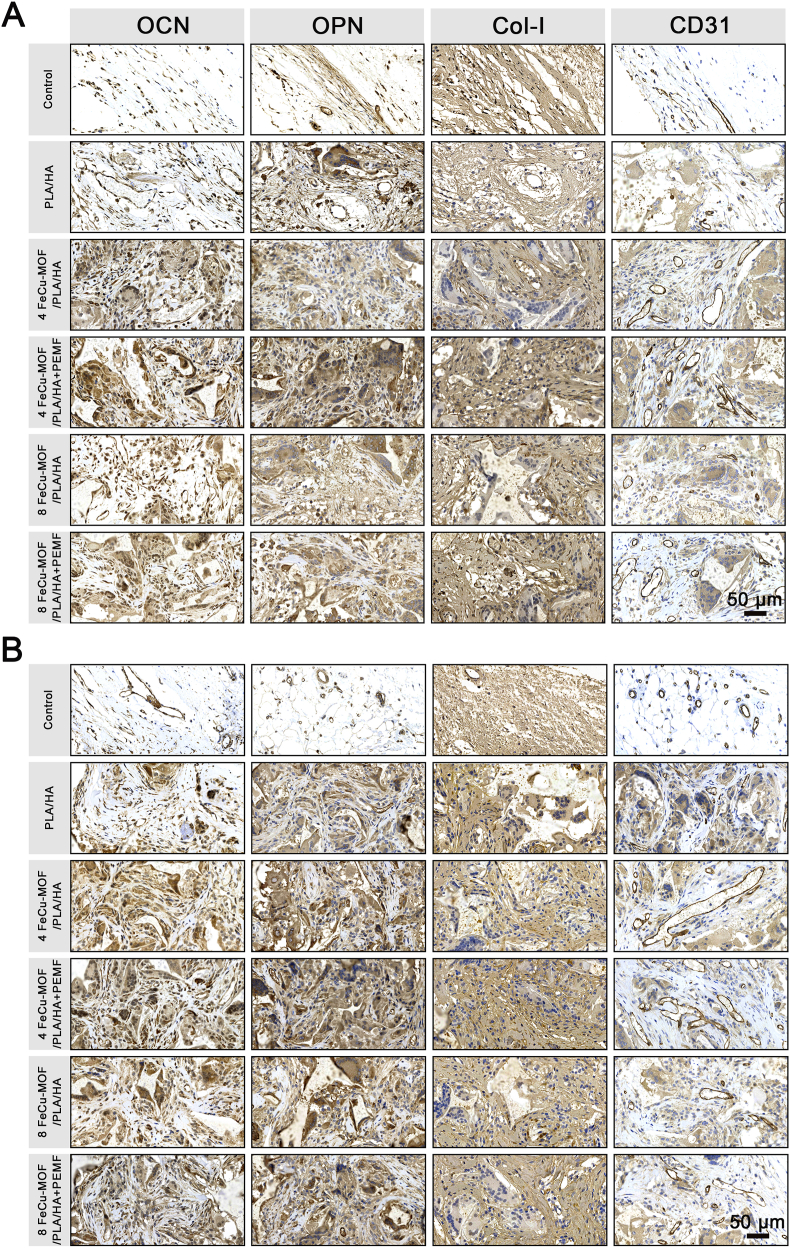
*In vivo* immunohistochemical analysis of osteogenic and angiogenic protein expression in the regenerated tissue. Representative immunohistochemical images of osteogenic markers OCN, OPN, and Col-I, along with the angiogenic marker CD31, at 4 weeks (A) and 8 weeks (B) post-implantation. Positive expression is indicated by brown staining. Compared to control groups, FeCu-MOF scaffolds markedly enhanced the expression of all markers. This effect was further amplified by PEMF stimulation, with the 4 % and 8 % FeCu-MOF + PEMF groups showing the most intense and widespread staining at 8 weeks. These results provide molecular-level evidence of coupled and enhanced osteogenesis and angiogenesis, corroborating the histological and micro-CT findings. Scale bar: 50 μm. (For interpretation of the references to color in this figure legend, the reader is referred to the Web version of this article.)

IHC staining for osteogenic markers (OPN, Col-Ⅰ, OCN) and the angiogenic marker (CD31) corroborated the histological findings (Fig. 9A and B). At both time points, expression levels of all these markers were markedly higher in the 4 % and 8 % FeCu-MOF groups, as well as in all PEMF-treated groups, compared to the Control and PLA/HA groups. When comparing PEMF-treated groups to their non-PEMF counterparts, PEMF stimulation consistently resulted in higher expression levels of OPN, OCN, Col-Ⅰ, and CD31. These results indicate that the combination of PEMF with the scaffold materials promoted enhanced osteogenesis and angiogenesis. Collectively, the IHC data align with the micro-CT and histological evidence, suggesting robust new bone formation and vascularization, particularly in the groups receiving both FeCu-MOF scaffolds and PEMF treatment.

## 4 Discussion

The effective repair of large bone defects remains a significant clinical hurdle, largely due to the suboptimal structural and functional characteristics of conventional grafts which limit their therapeutic efficacy [19,34]. This study introduces a chemophysical dual-responsive system to address these limitations, featuring a PLA/HA scaffold functionalized with a structurally engineered bimetallic FeCu-MOF. This MOF was designed for the programmed co-release of Fe^3+^ and Cu^2+^ ions and to achieve synergistic effects with PEMF stimulation. Our comprehensive findings demonstrate that this rationally designed system successfully integrates these dual stimuli. The engineered FeCu-MOF provided controlled ion release that fostered osteogenic and pro-angiogenic activity *in vitro*. Crucially, PEMF stimulation significantly amplified these cellular responses. This chemophysical synergy ultimately translated to markedly improved vascularized bone regeneration in a rat cranial defect model. These results highlight the therapeutic potential of such integrated approaches [35], inviting further discussion on the underlying structure-function relationships and synergistic mechanisms.

The engineered physical characteristics of the FeCu-MOF/PLA/HA scaffolds were crucial for creating a cell-conducive environment [36]. Their interconnected porosity (200–400 μm), intentionally designed via particulate leaching, mimics native bone architecture, thereby facilitating essential regenerative cellular processes [37,38]. Notably, the incorporation of FeCu-MOF significantly improved surface hydrophilicity and increased surface roughness. These MOF-induced surface modifications are known to promote protein adsorption, which in turn enhanced BMSCs adhesion, spreading, and proliferation [[39], [40], [41]], consistent with our biocompatibility findings. Such improved early cell-material interactions likely primed the BMSCs for subsequent differentiation cues from released ions and PEMF stimulation.

The programmed co-release of Fe^3+^ and Cu^2+^ ions, a key functional attribute engineered into the FeCu-MOF structure, is central to the scaffold's intrinsic bioactivity [42]. As shown by our release studies, the distinct release kinetics of these ions from the composite scaffold provide temporally relevant ionic signals crucial for guiding cellular responses. Specifically, the observed release profiles—a relatively faster initial Fe^3+^ liberation followed by sustained co-release of both Fe^3+^ and Cu^2+^—are designed to orchestrate a temporal sequence of biological cues. The initial burst of Fe^3+^ potentially supports early osteoblast proliferation and matrix production, an effect consistent with the upregulation of osteogenic markers [43,44]. Concurrently and subsequently, the sustained release of Cu^2+^ is anticipated to promote angiogenesis, a critical complementary process. The pro-angiogenic role of Cu^2+^, often mediated via HIF-1α stabilization, is well-established [45]. Although direct *in vitro* angiogenesis assays with the FeCu-MOF scaffolds were not conducted in this specific study arm, separate experiments provided relevant insights. These experiments, using analogous Cu-MOF nanoparticles (see Supporting Information Fig. S1), confirmed significant promotion of endothelial cell tube formation. Furthermore, these preliminary studies suggested PEMF treatment could potentially enhance this pro-angiogenic effect [25]. This programmed dual-ion release strategy is an inherent outcome of our bimetallic FeCu-MOF's engineered structure and composition. Our findings align with the state-of-the-art concept of using specific ion combinations to direct tissue regeneration, which was powerfully exemplified by recent work from Wang et al., demonstrating robust vascularized bone regeneration through the synergistic action of Mg^2+^ and Si^2+^ ions [16]. Together, these studies underscore the versatility and potency of 'ion-engineering' as a therapeutic strategy. Consequently, it provides an integrated, material-intrinsic approach to address the critical angiogenesis-osteogenesis coupling challenge [46,47]. Such an approach also holds the potential to simplify scaffold design compared to more complex multi-component or growth factor-based delivery systems [48], relying instead on the precisely controlled release from a single, functional MOF component.

A key finding of this study, validating our hypothesis of chemophysical synergy, is the significant synergistic interaction observed between the ion-releasing FeCu-MOF scaffold and PEMF stimulation. This approach aligns with a sophisticated paradigm in biomaterials engineering where external physical stimuli are harnessed to dynamically modulate cellular interactions. For instance, Li et al. elegantly demonstrated that an external electric field could stretch RGD-functionalized polymer chains to expose cryptic cell-adhesive motifs, thereby precisely controlling cell adhesion and dynamics [49]. Our findings build upon this concept, showing that PEMF acts as a potent non-invasive modulator that synergistically augments the biological effects of the ion-releasing scaffold. Our *in vitro* results consistently showed that PEMF treatment amplified BMSCs proliferation, ALP activity, and matrix mineralization beyond levels achieved by the scaffolds alone. This synergy suggests that PEMF enhances cellular responsiveness to the specific ionic milieu created by the engineered FeCu-MOF. While the precise mechanisms require further investigation, plausible explanations include PEMF-induced modulation of ion channels or mechanosensitive pathways, potentially leading to amplified crosstalk with ion-activated signaling (e.g., Wnt, HIF-1α) within the FeCu-MOF-conditioned microenvironment [24,50,51]. Regardless of the exact pathways, our results strongly indicate that PEMF acts as a potent non-invasive modulator that augments the biological effects of the ion-releasing scaffold. This synergistic enhancement is therapeutically significant, offering potential for improved efficacy or reduced ion dosages [52]. Crucially, it also underscores the profound value of integrating designed material-based chemical cues with physical stimuli for promoting enhanced tissue regeneration.

The robust *in vivo* efficacy of our rationally designed chemophysical system was decisively demonstrated in the rat cranial defect model, providing strong validation for its translational potential. Consistent with *in vitro* observations, the FeCu-MOF scaffolds alone, by virtue of their structure-derived ion release, significantly enhanced bone regeneration compared to controls. Crucially, the integration of PEMF stimulation elicited a pronounced synergistic effect *in vivo*. This synergistic effect was evidenced across multiple scales. Macroscopically, treatment resulted in more extensive new bone formation bridging the defect, along with significantly improved bone volume, density, and trabecular architecture. Histologically, more mature and well-integrated bone tissue was observed, seamlessly connected with the host bone. At the protein level, the PEMF-treated FeCu-MOF groups showed enhanced co-expression of key osteogenic (OPN, Col-Ⅰ, OCN) and angiogenic (CD31) markers within the defect site. This concurrent upregulation of these key proteins provides compelling evidence for promoted and coupled vascularization and osteogenesis – a cornerstone of effective bone repair – driven by the combined chemophysical stimuli.

A key finding from our quantitative micro-CT analysis was the significant reduction in Tb.Sp in PEMF-treated groups. This indicates the formation of a denser, more interconnected, and structurally superior bone matrix. Our histological data provide a clear mechanistic basis for this observation. The Masson's trichrome staining revealed that PEMF stimulation promoted the deposition of a more mature and densely organized collagen network. We postulate that the synergistic interaction between the scaffold's ionic cues and the PEMF field not only boosts overall osteogenic activity but also modulates the cellular processes governing extracellular matrix organization. This leads to a superiorly organized collagen template for mineralization, which ultimately manifests as a more compact trabecular architecture and a reduction in Tb.Sp.

These comprehensive *in vivo* findings strongly suggest that PEMF stimulation acts not merely as a simple amplifier of the ionic signals, but as a synergistic enhancer of the entire biological program orchestrated by the FeCu-MOF. The precisely timed Fe^3+^ and Cu^2+^ ionic cues, delivered by the engineered scaffold structure, likely augmented cellular responsiveness to PEMF. Concurrently, PEMF may have activated parallel or complementary signaling pathways that potentiate both osteogenesis and angiogenesis. Such activation could, in turn, render the effects initiated by ion release more pronounced, sustained, and effectively coupled. For instance, it is plausible that PEMF exerted a dual effect: amplifying Cu^2+^-induced HIF-1α expression while simultaneously promoting Fe^3+^-mediated upregulation of osteogenic genes. This combined action likely led to the observed robust and truly coupled angiogenic-osteogenic response.

The superior efficacy of our engineered chemophysical system evidently stems from a sophisticated interplay. This interplay involves material-derived chemical signals (programmed dual-ion release) and external physical signals (PEMF stimulation) converging to create a highly favorable regenerative microenvironment. This dual-pronged, synergistic approach efficiently drove comprehensive tissue regeneration, encompassing not only bone mineralization and maturation but also the concurrent formation of a robust neovascular network. Consequently, the system presented herein offers a more holistic and effective solution for achieving functional, vascularized bone regeneration, especially when compared to conventional single-factor or unimodal strategies. This is particularly relevant for challenging defects with compromised vascularity. This strategic interplay successfully establishes a robust framework for achieving angiogenic-osteogenic coupling. However, to translate this qualitative success into maximal therapeutic benefit, the quantitative aspects of ion release, specifically their concentration and dosage, become critically important.

Our systematic evaluation of varying FeCu-MOF concentrations highlighted a significant dose-dependent effect of the released Fe^3+^ and Cu^2+^ ions on modulating bone regeneration. Consistently, the 4 FeCu-MOF/PLA/HA composite scaffold exhibited superior biological effects across key *in vitro* metrics (BMSCs proliferation, ALP activity, matrix mineralization) and *in vivo* outcomes (new bone volume, vascular density). While the 8 % FeCu-MOF group still surpassed MOF-free controls in promoting key regenerative outcomes, a clear non-linear dose-response relationship was observed. Specifically, increasing the FeCu-MOF concentration beyond the previously identified optimal level of 4 % (to 8 %) did not lead to further enhancements. Instead, a plateau in efficacy, or even a slight decline, was generally observed. This strongly suggests the existence of a precise optimal therapeutic window for the released Fe^3+^ and Cu^2+^ ions. Within this optimal therapeutic window, the released ions can effectively stimulate cellular responses and foster angiogenic-osteogenic coupling. However, ion concentrations exceeding this range, such as those potentially delivered by the 8 % FeCu-MOF group, may elicit less favorable or even detrimental cellular responses. This aligns with literature reports indicating that excessively high concentrations of Fe^3+^ or Cu^2+^ can induce oxidative stress, disrupt cellular homeostasis, or exhibit direct cytotoxicity, thereby inhibiting cell proliferation, differentiation, or functional expression [53,54]. Therefore, the observed plateau or slight decline in performance for the 8 % group might represent an initial manifestation of this double-edged sword effect, where beneficial ionic concentrations transition towards inhibitory levels.

This observed non-linear dose-response holds significant implications for the rational design of ion-releasing biomaterials. It underscores a crucial principle: merely increasing the payload of bioactive ions does not invariably lead to superior therapeutic outcomes. Instead, precise engineering control over both the dose and the temporal profile of ion release is critical, perhaps even more so, for achieving optimal biological efficacy. This principle is particularly pertinent to our chemophysical synergistic strategy. Optimizing the chemical signaling component is a fundamental prerequisite for the physical stimulus (PEMF) to exert its maximal potentiating and synergistic effect. This optimization primarily involves controlling the concentration and release kinetics of ions derived from the engineered FeCu-MOF. Therefore, future investigations should focus on the finer structural engineering of the FeCu-MOF. Such engineering would include optimizing its loading within the scaffold and potentially tailoring its framework architecture—for example, by modifying porosity or ligand functionalization. The ultimate aim is to more accurately achieve and maintain this optimal ionic concentration window. Such structure-driven optimization will be crucial for maximizing the overall therapeutic efficacy of this combined chemophysical approach and translating its potential.

While this study demonstrates the promising potential of the engineered FeCu-MOF/PEMF system, several avenues warrant further investigation to fully realize its clinical applicability. Firstly, a deeper understanding of the precise molecular pathways underlying the observed chemophysical synergies is crucial. This would also include investigating the direct influence of PEMF on the ion release kinetics from the scaffold, which could provide further mechanistic insights into the observed synergy. Future studies could employ transcriptomic or proteomic analyses, potentially combined with pathway-specific inhibitors, to dissect these complex interactions. Secondly, to bridge the gap towards clinical translation, evaluating the system's performance in load-bearing large animal models under physiologically relevant mechanical conditions is essential. Such studies would also allow for a more comprehensive assessment of long-term bone remodeling, material degradation profiles, and extended biocompatibility. Thirdly, further optimization of PEMF parameters (e.g., frequency, intensity, duration) could potentially yield even greater therapeutic benefits. The success of our dual-responsive system resonates with the forefront of regenerative medicine aimed at creating multifunctional biomaterials that orchestrate complex tissue regeneration. A visionary example is the development of ‘dual-electroactive’ cryogel microspheres by Wang et al., which combine intrinsic piezoelectric and conductive properties with bioactive ion release [55]. Their system was shown to promote a holistic neurovascularized bone regeneration, encompassing not only angiogenesis and osteogenesis but also lymphogenesis and neurogenesis. This highlights the immense therapeutic potential of advanced platforms that integrate material-derived chemical signals with physical cues—whether externally applied like PEMF in our study, or intrinsically generated. It also opens exciting future avenues for our FeCu-MOF/PEMF platform, such as exploring its potential effects on neuro- and lymphangiogenic processes in bone repair. Nevertheless, despite these future directions, the current study provides a strong proof-of-concept. The study validates the FeCu-MOF + PEMF platform as an innovative and effective strategy for enhancing vascularized bone regeneration. The platform's design is based on the rational integration of structure-derived ionic cues with physical stimulation. This work highlights the significant potential of such engineered chemophysical synergistic systems in addressing complex tissue engineering challenges.

## 5 Conclusion

In summary, this study successfully developed and validated a novel chemophysical dual-responsive system for bone regeneration, based on PLA/HA composite scaffolds functionalized with a bimetallic FeCu-MOF specifically designed for controlled ion co-release. We demonstrated that this system enables the programmed delivery of Fe^3+^ and Cu^2+^ ions, which intrinsically promoted osteogenic differentiation and pro-angiogenic activity *in vitro*, and ultimately fostered coupled and vascularized bone formation *in vivo*. Crucially, integrating PEMF stimulation yielded significant synergistic effects, markedly amplifying these regenerative processes and leading to accelerated and enhanced bone regeneration in a rat cranial defect model. This work provides compelling evidence for the efficacy of combining tailored ion release from an advanced biomaterial with physical field modulation. Such an integrated, synergistic strategy offers a promising and innovative approach with considerable potential for clinical translation in challenging bone defect repair scenarios.

## CRediT authorship contribution statement

**Dongdong Guo:** Writing – original draft, Visualization, Validation, Methodology, Formal analysis, Data curation. **Wenjie Wang:** Methodology, Investigation, Formal analysis. **Dongyang Zhao:** Visualization, Software. **Tianyu Chen:** Validation, Resources. **Xingyu Ma:** Visualization, Methodology. **Yixiao Li:** Methodology, Data curation. **Xiaojun Zhang:** Writing – review & editing, Visualization, Supervision, Project administration, Funding acquisition, Conceptualization.

## Declaration of competing interest

The authors declare that they have no known competing financial interests or personal relationships that could have appeared to influence the work reported in this paper.

## Data Availability

Data will be made available on request.
