# Supplementary material for: Synergistic dual chemophysical FeCu-MOF scaffold with PEMF stimulation drives angiogenic-osteogenic coupling for bone regeneration
Source: Mater Today Bio. 2025 Sep 18;35:102324. doi: 10.1016/j.mtbio.2025.102324 (PMC12489837; doi:10.1016/j.mtbio.2025.102324)
Supplement: Multimedia component 1 [file mmc1.docx]

**Supporting Information**

**Synergistic dual chemophysical FeCu-MOF scaffold with PEMF stimulation drives angiogenic-osteogenic coupling for bone regeneration**

*Dongdong Guo*^1^*^,^*^2^*, Wenjie Wang*^1,2^*, Dongyang Zhao*^1^*, Tianyu Chen*^2^*, Xingyu Ma*^1^*, Yixiao Li*^1^*, Xiaojun Zhang*^1^*

^1^ School of Medicine, Northwest University, Xi’an, Shaanxi Province,710069, China;

^2^ Key Laboratory of Resource Biology and Biotechnology Western China, Ministry of Education; Provincial Key Laboratory of Biotechnology, College of Life Sciences, Northwest University, Xi’an, Shaanxi Province,710069, China;

*Correspondence author’s E-mail: [zhangxj@nwu..edu.cn](mailto:zhangxj@nwu..edu.cn)

**1. Supplementary methods**

**1.1. Synthesis and characterization of Cu-MOF-74**

Cu-MOF-74 was synthesized and characterized according to our previously reported method [1]. Briefly, 0.4 g of Cu(OAc)_2_·H_2_O was dissolved in 10 mL of methanol to obtain a 40 g/L solution. Then, 0.2 g of DHTP was added to 5 mL of methanol, followed by the dropwise addition of the Cu(OAc)_2_·H_2_O solution. The mixture was magnetically stirred for 24 h, and the resulting reddish-brown crystalline solid was collected by filtration. The resulting Cu-MOF-74 was washed with methanol four times to remove impurities and dried in a vacuum at 85 ℃ for 24 h. The microstructure of Cu-MOF-74 was observed using scanning electron microscopy (SEM; Hitachi, S-4800, Tokyo, Japan) and transmission electron microscopy (TEM; JEOL, JEM-2100Plus, Tokyo, Japan). The crystal structures of the synthesized nanoparticles were analyzed using X-ray diffraction (XRD; Bruker-D8, Bremen, Germany). Fourier-transform infrared (FTIR; AVATAR-FTIR-360, Thermo Fisher Scientific, Waltham, MA, USA) spectra were recorded in the range of 4000–400 cm^−1^. Elemental mapping of Cu-MOF-74 was conducted using an energy-dispersive spectrometer(EDS; Bruker QUANTAX 400, Bremen, Germany).

**1.2.** **Endothelial cell culture**

In this study, bone marrow-derived endothelial cells were isolated from the femurs and tibias of 6-7 day-old Sprague-Dawley rat pups. The isolation procedure was as follows: Under aseptic conditions, femurs and tibias were excised. The bone marrow was flushed from the bone cavities using a 1 mL sterile syringe with phosphate-buffered saline (PBS) containing penicillin (100 IU/mL) and streptomycin (100 µg/mL). The bone marrow suspension was carefully layered onto an equal volume of rat lymphocyte separation medium (density 1.083 g/mL) in a centrifuge tube, ensuring a clear interface. The suspension was centrifuged at 2,500 rpm for 30 min at room temperature. After centrifugation, the mononuclear cell layer located at the interface between the plasma and separation medium layers was carefully collected using a Pasteur pipette and transferred to a new centrifuge tube containing PBS. The collected cells were pelleted by centrifugation at 1500 rpm for 10 min. The cell precipitate was resuspended in endothelial cell growth medium (ScienCell, USA) supplemented with 5% fetal bovine serum, 1% endothelial cell growth supplement, penicillin (100 IU/mL), and streptomycin (100 µg/mL). Cells were cultured at 37 °C in a humidified incubator with 5% CO_2_. The medium was replaced every 2-3 days. When primary endothelial cells reached 80-90% confluence, they were detached using 0.05% Trypsin-EDTA solution and passaged. Cells from passages 2-5 were used for experiments.

**1.3. *In vitro* tube formation assay**

To evaluate the pro-angiogenic potential, an *in vitro* tube formation assay was performed using Matrigel® Basement Membrane Matrix (Xiamen Mogengel, Xiamen, China). Briefly, pre-chilled 96-well plates were coated with 50 μL of Matrigel per well and allowed to polymerize at 37 °C for 30 min. BMECs, previously cultured as described, were harvested, counted, and resuspended in completely endothelial medium for the assay. Cells (2.0 × 10⁴ cells per well) were then seeded onto the polymerized Matrigel.

**Treatment Groups**: Cells were treated with conditioned media derived from composite scaffolds. Composite scaffolds (PCL/HA) containing varying weight percentages of Cu-MOF-74 (0%, 0.05%, 0.2%, or 1% w/w relative to the total scaffold mass) were fabricated. Extracts were prepared in accordance with the Chinese National Standard GB/T 16886.12-2017 (Biological evaluation of medical devices -- Part 12: Sample preparation and reference materials). Briefly, 1 g of scaffold material was incubated per 10 mL of basal endothelial medium for 24 h at 37 °C. The supernatant was collected, filtered (0.22 µm), and used as conditioned medium. These conditioned media were designated as 0 Cu, 0.05 Cu, 0.2 Cu, and 1 Cu, respectively. The 0 Cu group (extract from scaffolds without Cu-MOF-74) served as the negative control.

**PEMF Treatment**: For PEMF treatment, cell culture plates were exposed daily to a pulsed electromagnetic field (3.6 mT, 16 Hz, 60 min per exposure) using a stimulator (GS-100A, Jinan Chuangbo Technology Co., Ltd., Shandong, China). Control plates (0 mT, sham exposure) were placed in an identical but inactive device for the same duration.

At 12 h post-seeding, tube formation was observed and imaged using an inverted phase-contrast microscope.

**1.4. Quantitative analysis of tube formation**

Microscopic images captured at the 12-hour time point were used for quantitative analysis. Images from 3-5 randomly selected fields per well were analyzed using ImageJ software equipped with the Angiogenesis Analyzer plugin. Parameters quantified included total tube length and the number of branching points (nodes). Data were obtained from three independent experiments.

**1.5. Statistical Analysis**

Quantitative data are expressed as mean ± standard error of the mean (SEM). Statistical comparisons between multiple groups were performed using One-way ANOVA followed by Specify post-hoc test. A *p*-value < 0.05 was considered statistically significant. Comparisons between PEMF-treated and non-treated groups at the same Cu-MOF concentration were made using ANOVA.

**2. Supplementary results**

**2.1. Cu-MOF-74 and PEMF promote** **endothelial cells tube formation *in vitro***

To investigate the pro-angiogenic effects of Cu-MOF-74 and its interaction with PEMF stimulation, we assessed the tube formation capacity of rat endothelial cells on Matrigel. Representative phase-contrast microscopy images at 12 h post-seeding are shown in Fig. S1A. In the absence of Cu-MOF-74 extract (0 Cu group, no PEMF), endothelial cells formed a limited and poorly organized tubular network. The addition of extracts from scaffolds containing lower concentrations of Cu-MOF-74 (0.05 Cu and 0.2 Cu groups) dose-dependently promoted the formation of more extensive and interconnected capillary-like structures compared to the 0 Cu control. However, the extract from scaffolds with the highest Cu-MOF-74 concentration (1 Cu group) appeared to disrupt network formation and potentially affect cell morphology, suggesting a concentration-dependent effect with an optimal window.

PEMF stimulation alone (0 Cu + PEMF group) showed a slight enhancement in tube formation compared to the sham control (0 Cu, 0 mT). Notably, when combined with effective concentrations of Cu-MOF-74 extract (0.05 Cu and 0.2 Cu), PEMF treatment consistently resulted in more robust and complex network formation compared to the corresponding non-PEMF groups (0 mT) at the same Cu-MOF-74 extract concentration (Fig. S1A).

Quantitative analysis of total tube length and number of branching points at 12 h (Fig. S1B) confirmed these observations. Both 0.05 Cu and 0.2 Cu groups (without PEMF) significantly increased tube length and node number compared to the 0 Cu control (without PEMF). The 1 Cu group showed significantly reduced network formation compared to the optimal concentrations (0.05 Cu and 0.2 Cu). Importantly, PEMF treatment significantly enhanced both total tube length and node number in the presence of 0.05 Cu and 0.2 Cu extracts compared to their respective 0 mT counterparts, indicating a synergistic or additive pro-angiogenic effect between Cu-MOF-74 extracts and PEMF stimulation *in vitro*. These findings support the hypothesis that the copper component (from Cu-MOF-74) contributes to the pro-angiogenic potential observed in the FeCu-MOF system and that PEMF can further enhance this effect.


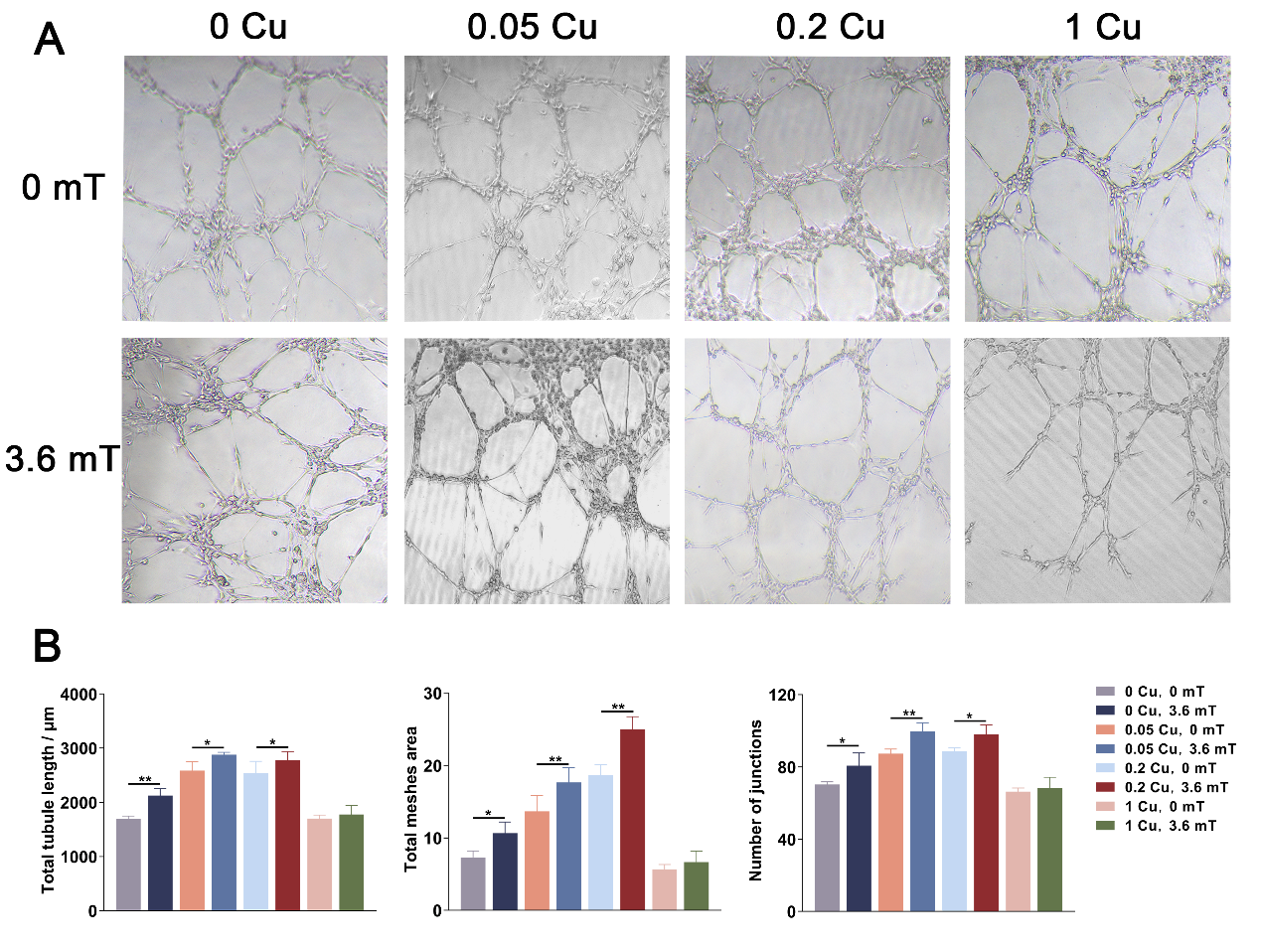


**Fig. S1**. In vitro tube formation assay of endothelial cells treated with Cu-MOF-74 and PEMF.

1. Representative phase-contrast microscopy images of rat endothelial cells tube formation on Matrigel after 12 h of incubation with extracts derived from scaffolds containing different concentrations of Cu-MOF-74 (0%, 0.05%, 0.2%, 1% w/w in scaffold, denoted as 0 Cu, 0.05 Cu, 0.2 Cu, 1 Cu respectively) with or without PEMF stimulation (0 mT or 3.6 mT, 16 Hz, 60 min). (B) Quantitative analysis of total tube length and number of branching points per field at the 12-hour time point. Data are presented as mean ± SEM (n=3). PEMF-treated groups were compared to their corresponding untreated (0 mT) groups at the same Cu-MOF-74 extract concentration: **p* < 0.05, ***p* < 0.01.

**Reference**

[1] Zhu T, Ni Q, Wang W, Guo D, Li Y, Chen T, Zhao D, Ma X, Zhang X. Cu-MOF-Decorated 3D-Printed Scaffolds for Infection Control and Bone Regeneration. J Funct Biomater. 2025;16(3):83. https://doi.org/10.3390/jfb16030083.
